# Exploring the impact of housing insecurity on the health and wellbeing of children and young people in the United Kingdom: a qualitative systematic review

**DOI:** 10.1186/s12889-024-19735-9

**Published:** 2024-09-09

**Authors:** Emma S. Hock, Lindsay Blank, Hannah Fairbrother, Mark Clowes, Diana Castelblanco Cuevas, Andrew Booth, Amy Clair, Elizabeth Goyder

**Affiliations:** 1 Sheffield Centre for Health and Related Research, University of Sheffield, Sheffield, UK; 2 Australian Centre for Housing Research, University of Adelaide, Adelaide, Australia

**Keywords:** Systematic review, Housing insecurity, Housing instability, Children, Adolescents, Young people, Health, Wellbeing

## Abstract

**Background:**

Housing insecurity can be understood as experiencing or being at risk of multiple house moves that are not through choice and related to poverty. Many aspects of housing have all been shown to impact children/young people’s health and wellbeing. However, the pathways linking housing and childhood health and wellbeing are complex and poorly understood.

**Methods:**

We undertook a systematic review synthesising qualitative data on the perspectives of children/young people and those close to them, from the United Kingdom (UK). We searched databases, reference lists, and UK grey literature. We extracted and tabulated key data from the included papers, and appraised study quality. We used best fit framework synthesis combined with thematic synthesis, and generated diagrams to illustrate hypothesised causal pathways.

**Results:**

We included 59 studies and identified four populations: those experiencing housing insecurity in general (40 papers); associated with domestic violence (nine papers); associated with migration status (13 papers); and due to demolition-related forced relocation (two papers). Housing insecurity took many forms and resulted from several interrelated situations, including eviction or a forced move, temporary accommodation, exposure to problematic behaviour, overcrowded/poor-condition/unsuitable property, and making multiple moves. Impacts included school-related, psychological, financial and family wellbeing impacts, daily long-distance travel, and poor living conditions, all of which could further exacerbate housing insecurity. People perceived that these experiences led to mental and physical health problems, tiredness and delayed development. The impact of housing insecurity was lessened by friendship and support, staying at the same school, having hope for the future, and parenting practices. The negative impacts of housing insecurity on child/adolescent health and wellbeing may be compounded by specific life circumstances, such as escaping domestic violence, migration status, or demolition-related relocation.

**Conclusion:**

Housing insecurity has a profound impact on children and young people. Policies should focus on reducing housing insecurity among families, particularly in relation to reducing eviction; improving, and reducing the need for, temporary accommodation; minimum requirements for property condition; and support to reduce multiple and long-distance moves. Those working with children/young people and families experiencing housing insecurity should prioritise giving them optimal choice and control over situations that affect them.

**Supplementary Information:**

The online version contains supplementary material available at 10.1186/s12889-024-19735-9.

## Introduction

The impacts of socioeconomic position in childhood on adult health outcomes and mortality are well documented in quantitative analyses (e.g., [1]). Housing is a key mechanism through which social and structural inequalities can impact health [2]. The impact of housing conditions on child health are well established [3]. Examining the wellbeing of children and young people within public health overall is of utmost importance [4]. Children and young people (and their families) who are homeless are a vulnerable group with particular difficulty in accessing health care and other services, and as such, meeting their needs should be a priority [5].

An extensive and diverse evidence base captures relationships between housing and health, including both physical and mental health outcomes. Much of the evidence relates to the quality of housing and specific aspects of poor housing including cold and damp homes, poorly maintained housing stock or inadequate housing leading to overcrowded accommodation [6–13]. The health impacts of housing insecurity, together with the particular vulnerability of children and young people to the effects of not having a secure and stable home environment, continue to present a cause for increased concern [7, 8, 11, 14]. The National Institute for Health and Care Research (NIHR) Public Health Reviews (PHR) Programme commissioned the current review in response to concerns about rising levels of housing insecurity and the impact of housing insecurity on the health and wellbeing of children and young people in the United Kingdom (UK).

### Terminology and definitions related to housing insecurity

Numerous diverse terms are available to define housing insecurity, with no standard definition or validated instrument. For the purpose of our review, we use the terminology and definitions used by the Children’s Society, which are comprehensive and based directly on research with children that explores the relationship between housing and wellbeing [15]. They use the term “housing insecurity” for *those experiencing and at risk of multiple moves that are (i) not through choice and (ii) related to poverty* [15]. This reflects their observation that multiple moves may be a positive experience if they are by choice and for positive reasons (e.g., employment opportunities; moves to better housing or areas with better amenities). This definition also acknowledges that the wider health and wellbeing impacts of housing insecurity may be experienced by families that may not have experienced frequent moves but for whom a forced move is a very real possibility. The Children’s Society definition of housing insecurity encompasses various elements (see Table 1).

**Table 1:** Elements of housing insecurity encompassed in the Children’s Society Definition

Element	Definition
Housing instability [16, 17]	Having difficulty paying rent, having frequent moves, living in overcrowded conditions, or doubling up with friends and relatives
Unstable or precarious housing [18]	Living somewhere that does not provide a sense of safety and security. Includes homelessness and/or precarious living circumstances
Financial insecurity [17]	Spending more than 50% of household income on housing [17]
Spatial insecurity [19]	The inability to remain in a given dwelling or wider neighbourhood area, including through eviction and forced moves
Relational insecurity [19]	The ways in which individuals’ experiences of housing and home are bound up with relationships with others
Residential mobility [20, 21]	The frequency and/or number or distance of moves [20], in particular, residential transience (a high frequency of moves) [21]

### Housing insecurity in the UK today – the extent of the problem

Recent policy and research reports from multiple organisations in the UK highlight a rise in housing insecurity among families with children [19, 22, 23]. Housing insecurity has grown following current trends in the cost and availability of housing, reflecting in particular the rapid increase in the number of low-income families with children in the private rental sector [19, 22, 24], where housing tenures are typically less secure. The ending of a tenancy in the private rental sector was the main cause of homelessness given in 15,500 (27% of claims) of applications for homelessness assistance in 2017/18, up from 6,630 (15% of claims) in 2010/11 for example [25]. The increased reliance on the private rented sector for housing is partly due to a lack of social housing and unaffordability of home ownership [23]. The nature of tenure in the private rental sector and gap between available benefits and housing costs means even low-income families that have not experienced frequent moves may experience the negative impacts of being at persistent risk of having to move [26]. Beyond housing benefit changes, other changes to the social security system have been linked with increased housing insecurity. The roll-out of Universal Credit^1^, with its built-in waits for payments, has been linked with increased rent arrears [27, 28]. The introduction of the benefit cap, which limits the amount of social security payments a household can receive, disproportionately affects housing support and particularly affecting lone parents [29–31].

The increase in families experiencing housing insecurity, including those living with relatives or friends (the ‘hidden homeless’) and those in temporary accommodation provided by local authorities, are a related consequence of the lack of suitable or affordable rental properties, which is particularly acute for lone parents and larger families. The numbers of children and young people entering the social care system or being referred to social services because of family housing insecurity contributes further evidence on the scale and severity of the problem [32].

The COVID-19 pandemic exacerbated housing insecurity in the UK [24], with the impacts continuing to be felt. In particular, the pandemic increased financial pressures on families (due to loss of income and increased costs for families with children/young people at home). These financial pressures were compounded by a reduction in informal temporary accommodation being offered by friends and family due to social isolation precautions [24]. Further, the COVID-19 pandemic underscored the risks to health posed by poor housing quality (including overcrowding) and housing insecurity [24, 33]. Recent research with young people in underserved communities across the country also highlighted their experience of the uneven impact of COVID-19 for people in contrasting housing situations [34].

While the temporary ban on bailiff-enforced evictions, initiated due to the pandemic, went some way towards acknowledging the pandemic’s impact on housing insecurity, housing organisations are lobbying for more long-term strategies to support people with pandemic-induced debt and rent-arrears [33]. The Joseph Rowntree Foundation has warned of the very real risk of a ‘two-tier recovery’ from the pandemic, highlighting the ‘disproportionate risks facing people who rent their homes’ ([35], para. 1). Their recent large-scale survey found that one million renting households worry about being evicted in the next three months, and half of these were families with children [35]. The survey also found that households with children, renters from ethnic minority backgrounds and households on low incomes are disproportionately affected by pandemic-induced debt and rent arrears [35].

The cost-of-living crisis is exacerbating the impact of the COVID-19 pandemic, with many households experiencing or set to experience housing insecurity due to relative reductions in income accompanying increases in rent and mortgage repayments [36]. People experiencing or at risk of housing insecurity are disproportionately affected, due to higher food and utility costs [37].

### Research evidence on relationships between housing in childhood and health

Housing is a key social determinant of health, and a substantive evidence base of longitudinal cohort studies and intervention studies supports a causal relationship between the quality, affordability and stability of housing and child health [38]. Evidence includes immediate impacts on mental and physical health outcomes and longer-term life course effects on wider determinants of health including education, employment and income as well as health outcomes [39].

The negative health impact of poor physical housing conditions has been well documented [40, 41]. Housing instability and low housing quality are associated with worse psychological health among young people and parents [42, 43]. The UK National Children’s Bureau [22] draws attention to US-based research showing that policies that reduced housing insecurity for young children can help to improve their emotional health [44], and that successful strategies for reducing housing insecurity have the potential to reduce negative outcomes for children with lived experience of housing insecurity, including emotional and behavioural problems, lower academic attainment and poor adult health and wellbeing [45]. A variety of pathways have been implicated in the relationship between housing insecurity and child health and wellbeing, including depression and psychological distress in parents, material hardships and difficulties in maintaining a good bedtime routine [38]. Frequent moves are also associated with poorer access to preventive health services, reflected, for example, in lower vaccination rates [46, 47].

Housing tenure, unstable housing situations and the quality or suitability of homes are inter-related [48]. For example, if families are concerned that if they lost their home they would not be able to afford alternative accommodation, they may be more likely to stay in smaller or poor-quality accommodation or in a neighbourhood where they are further from work, school or family support. In this way, housing insecurity can lead to diverse negative health and wellbeing impacts relating to housing and the neighbourhoods, even if in the family does not experience frequent moves or homelessness [49]. Thus, the relationship between housing insecurity and child health is likely to be complicated by the frequent coexistence of poor housing conditions or unsuitable housing with housing insecurity. The relationship between unstable housing situations and health outcomes is further confounded by other major stressors, such as poverty and changes in employment and family structure, which may lead to frequent moves.

The evidence from cohort studies that show a relationship between housing insecurity, homelessness or frequent moves in childhood and health related outcomes can usefully quantify the proportion of children/young people and families at risk of poorer health associated with housing instability. It can, however, only suggest plausible causal associations. Further, the ‘less tangible aspects of housing’ such as instability are poorly understood [40]. Additional (and arguably stronger) evidence documenting the relationship between housing insecurity and health/wellbeing comes from the case studies and qualitative interviews with children and young people and families that explore the direct and indirect impacts of housing insecurity on their everyday lives and wellbeing. Thus, the current review aimed to identify, appraise and synthesise research evidence that explores the relationship between housing insecurity and the health and wellbeing among children and young people. We aimed to highlight the relevant factors and causal mechanisms to make evidence-based recommendations for policy, practice and future research priorities.

## Methods

We undertook a systematic review synthesising qualitative data, employing elements of rapid review methodology in recognition that the review was time-constrained. This involved two steps: (1) a single screening by one reviewer of titles and abstracts, with a sample checked by another reviewer; and (2) a single data extraction and quality assessment, with a sample checked by another reviewer) [50–52]. The protocol is registered on the PROSPERO registry, registration number CRD42022327506.

### Search strategy

Searches of the following databases were conducted on 8th April 2022 (from 2000 to April 2022): MEDLINE, EMBASE and PsycINFO (via Ovid); ASSIA and IBSS (via ProQuest) and Social Sciences Citation Index (via Web of Science). Due to the short timescales for this project, searches aimed to balance sensitivity with specificity, and were conceptualised around the following concepts: (housing insecurity) and (children or families) and (experiences); including synonyms, and with the addition of a filter to limit results to the UK where available [53]. To expedite translation of search strings across different databases, searches prioritised free text search strings (including proximity operators), in order to retrieve relevant terms where they occurred in titles, abstracts or any other indexing field (including subject headings). The searches of ASSIA and IBSS (via ProQuest) and Social Sciences Citation Index (via Web of Science) used a simplified strategy adapted from those reproduced in Additional File 1. Database searching was accompanied by scrutiny of reference lists of included papers and relevant systematic reviews (within search dates), and grey literature searching (see Supplementary Table 1, Additional File 2), which was conducted and documented using processes outlined by Stansfield et al*.* [54].

### Inclusion criteria

We included qualitative studies, including qualitative elements of mixed methods studies from published and grey literature (excluding dissertations and non-searchable books), that explored the impact of housing insecurity, defined according to the Children’s Society [15] definition (which includes actual or perceived insecurity related to housing situations), on immediate and short-term outcomes related to childhood mental and physical health and wellbeing (up to the age of 16), among families experiencing / at risk of housing insecurity in the UK (including low-income families, lone-parent families, and ethnic minority group families including migrants, refugees and asylum seekers). Informants could include children and young people themselves, parents / close family members, or other informants with insight into the children and young people’s experiences. Children and young people outside a family unit (i.e., who had left home or were being looked after by the local authority) and families from Roma and Irish Traveller communities were excluded, as their circumstances are likely to differ substantially from the target population.

### Study selection

Search results from electronic databases were downloaded to a reference management application (EndNote). The titles and abstracts of all records were screened against the inclusion criteria by one of three reviewers and checked for agreement by a further reviewer. Full texts of articles identified at abstract screening were screened against the inclusion criteria by one reviewer. A proportion (10%) of papers excluded at the full paper screening stage were checked by a second reviewer. Any disagreements were resolved through discussion.

Grey literature searches and screening were documented in a series of tables [54]. One reviewer (of two) screened titles of relevant web pages and reports against the inclusion criteria for each web platform searched, and downloaded and screened the full texts of potentially eligible titles. Queries relating to selection were checked by another reviewer, with decisions discussed among the review team until a consensus was reached.

One reviewer (of two) screened reference lists of included studies and relevant reviews for potentially relevant papers. One reviewer downloaded the abstracts and full texts of relevant references and assessed them for relevance.

### Data extraction

We devised a data extraction form based on forms that the team has previously tested for similar reviews of public health topics. Three reviewers piloted the extraction form and suggested revisions were agreed before commencing further extraction. Three reviewers extracted and tabulated key data from the included papers and grey literature sources, with one reviewer completing data extraction of each study and a second reviewer formally checking a 10% sample for accuracy and consistency. The following data items were extracted: author and year, location, aims, whether housing insecurity was an aim, study design, analysis, who the informants were, the housing situation of the family, reasons for homelessness or housing insecurity, conclusion, relevant policy/practice implications and limitations. Any qualitative data relating to housing insecurity together with some aspect of health or wellbeing in children and young people aged 0–16 years were extracted, including authors’ themes (to provide context), authors’ interpretations, and verbatim quotations from participants. We sought to maintain fidelity to author and participant terminologies and phrasing throughout.

### Quality appraisal

Peer-reviewed academic literature was appraised using the Critical Appraisal Skills Programme (CASP) checklist for qualitative studies [55] and the quality of grey literature sources (webpages and reports) was appraised using the Authority, Accuracy, Coverage, Objectivity, Date, Significance (AACODS) checklist [56]. Because of concerns about the lack of peer review and/or the absence of a stated methodology, it was decided to use the AACODS tool that extends beyond simple assessment of study design. A formal quality assessment checklist was preferred for journal articles that passed these two entry criteria. One reviewer performed quality assessment, with a second reviewer formally checking a 10% sample for accuracy and consistency.

### Development of the conceptual framework

**Fig. 1:**
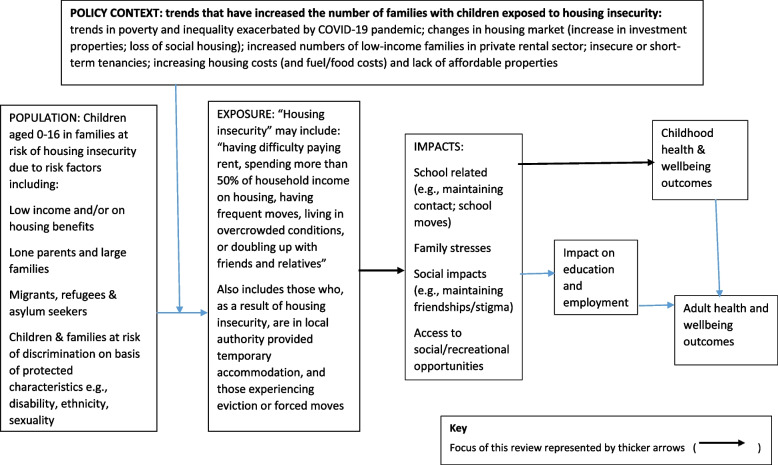
A priori conceptual framework for the relationship between housing insecurity and the health and wellbeing of children and young people

Prior to undertaking the current review, we undertook preliminary literature searches to identify an appropriate conceptual framework or logic model to guide the review and data synthesis process. However, we were unable to identify a framework that specifically focused on housing insecurity among children and young people and that was sufficiently broad to capture relevant contexts, exposures and impacts. We therefore developed an a priori conceptual framework based on consultation with key policy and practice stakeholders and topic experts and examination of key policy documents (see Fig. 1).

We initially consulted policy experts who identified relevant organisations including research centres, charities and other third sector organisations. We obtained relevant policy reports from organisational contacts and websites, including Child Poverty Action Group (CPAG), Crisis, Joseph Rowntree Foundation (JRF) and HACT (Housing Association Charitable Trust), NatCen (People Living in Bad Housing, 2013), the UK Collaborative Centre for Housing Evidence (CaCHE), and the Centre on Household Assets and Savings Management (CHASM) (Homes and Wellbeing, 2018). We also identified a key report on family homelessness from the Children’s Commissioner (Bleak Houses. 2019) and a joint report from 11 charities and advocacy organisations published by Shelter (Post-Covid Policy: Child Poverty, Social Security and Housing, 2022). We also consulted local authority officers with responsibility for housing and their teams in two local councils and third sector providers of housing-related support to young people and families (Centrepoint). Stakeholders and topic experts were invited to comment on the potential focus of the review and the appropriate definitions and scope for the ‘exposure’ (unstable housing), the population (children and young people) and outcomes (health and wellbeing). Exposures relate to how children and families experience housing insecurity, impacts are intermediate outcomes that may mediate the effects of housing insecurity on health and wellbeing (e.g., the psychological, social, and environmental consequences of experiencing housing insecurity), and outcomes are childhood health and wellbeing effects of housing insecurity (including the effects of the impacts/intermediate outcomes).

The contextual factors and main pathways between housing-related factors and the health and wellbeing of children and young people identified were incorporated into the initial conceptual framework. We then used this conceptual framework to guide data synthesis.

### Data synthesis

We adopted a dual approach whereby we synthesised data according to the a priori conceptual framework and sought additional themes, categories and nuance inductively from the data, in an approach consistent with the second stage of ‘best fit framework synthesis’ [57, 58]. We analysed inductive themes using the Thomas and Harden [59] approach to thematic synthesis, but coded text extracts (complete sentences or clauses) instead of coding line by line [60, 61].

First, one reviewer (of two) coded text extracts inductively and within the conceptual framework, simultaneously, linking each relevant text extract to both an inductive code based on the content of the text extract, and to an element of the conceptual framework. We assigned multiple codes to some extracts, and the codes could be linked to any single element or to multiple elements of the conceptual framework. During the process of data extraction, we identified four distinct populations, and coded (and synthesised) data discretely for each population. We initially coded data against the ‘exposure’, ‘impacts’ and ‘outcomes’ elements of the conceptual framework, however we subsequently added a further element within the data; ‘protective factors’. One reviewer then examined the codes relating to each element of the conceptual framework and grouped the codes according to conceptual similarity and broader meaning, reporting the thematic structure and relationships between concepts apparent from the text extracts both narratively and within a diagram to illustrate hypothesised causal pathways within the original conceptual framework, to highlight links between specific exposures, impacts and outcomes for each population. While we synthesised the findings by population initially, and present separate diagrams for each population, we present overall findings in this manuscript due to several similarities and then highlight any important differences for the domestic violence, migrant/refugee/asylum seeker, and relocation populations.

## Results

### Study selection and included studies

**Fig. 2:**
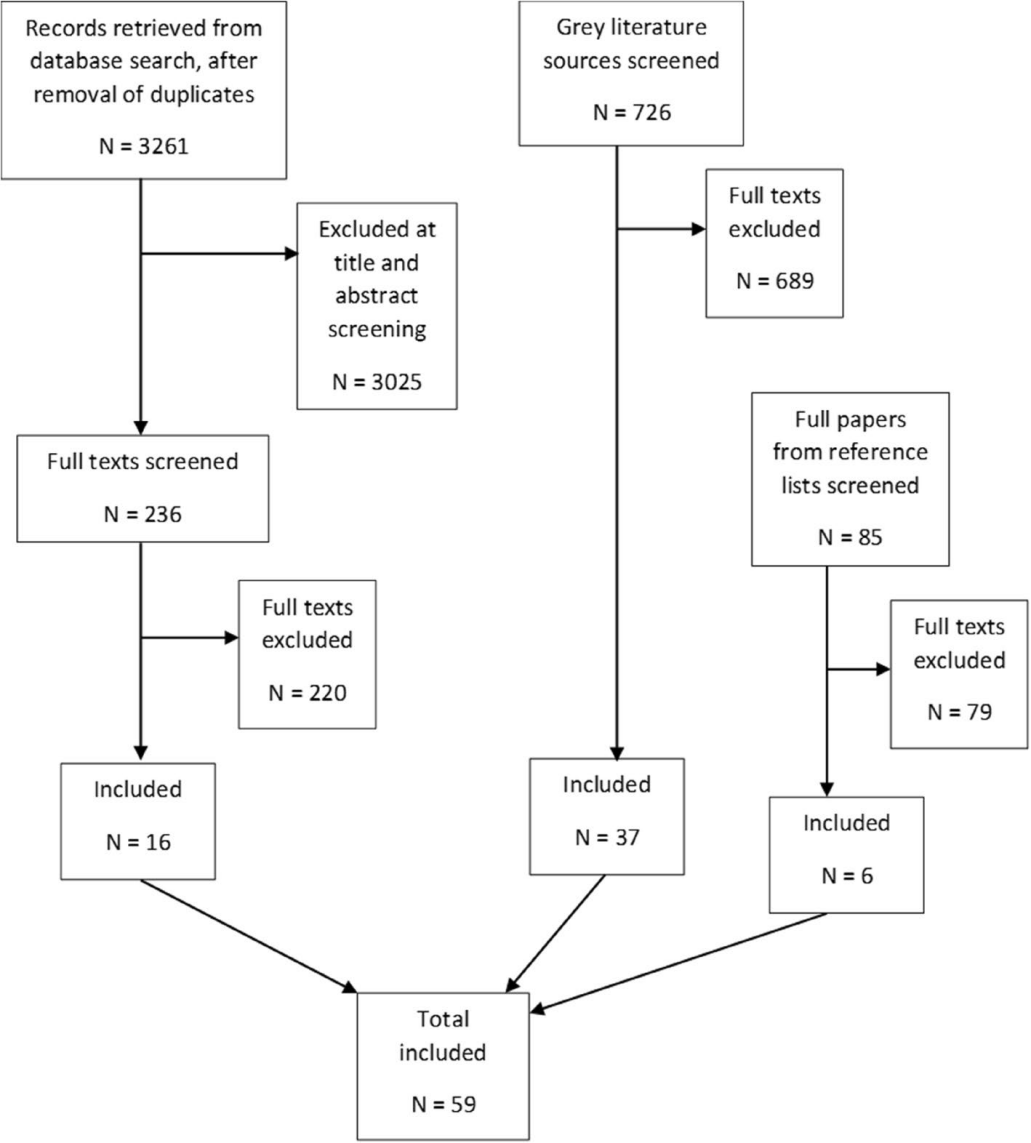
Flow diagram of study selection

Here we report the results of our three separate searchers. First, the database searches generated 3261 records after the removal of duplicates. We excluded 3025 records after title and abstract screening, examined 236 full texts, and included 16 peer-reviewed papers (reporting on 16 studies). The reasons for exclusion of each paper are provided in the Supplementary Table 2, Additional File 3. Second, we examined 726 grey literature sources (after an initial title screen) and included 37 papers. Third, we examined 85 papers that we identified as potentially relevant from the references lists of included papers and relevant reviews, and included six (two of which were peer-reviewed publications). Figure 2 summarises the process of study selection and Table 2 presents a summary of study characteristics. Of the included studies, 16 took place across the UK as a whole, one was conducted in England and Scotland, one in England and Wales and 17 in England. In terms of specific locations, where these were reported, 13 were reported to have been conducted in London (including specific boroughs or Greater London), two in Birmingham, one in Fife, two in Glasgow, one in Leicester, one in Rotherham and Doncaster, and one in Sheffield. The location of one study was not reported (Table 2).

**Table 2:** Study characteristics of included studies

**Study**	**Location**	HI an aim?* (Y/N)	**Population (numbers, where given)**	**Children (numbers, where given)**	**Informant/s (numbers, where given)**	**Study design Analysis**	**Housing situation of family**	**Reasons for homelessness/HI**
Backett-Milburn 2003 [62]	Scotland	N	General *N* = 15 children / parent-child dyads	15 children, aged 9-12 years. Only one reported on housing.	Children and their parents	Semi-structured interviews using child-appropriate techniques. Thematic analysis.	Vulnerably housed	Unemployment of parents
Bowyer 2015 [63]	Unclear	Y	Domestic violence *N* = 5 children, girls aged 10-16 exposed to domestic violence	5 children	Children	Semi-structured interviews. Interpretative phenomenological analysis	Temporary accommodation, mostly refuges	Domestic violence
Bradley 2020 [64]	London	N	General *N* = 13 parents, living in temporary accommodation	Numbers not reported. Families had 1 to 4 children. Aged 2 to 9 years (mean 3.6 years).	Parents	Semi-structured interviews. Thematic analysis.	Temporary accommodation	Not reported
Coram Children’s Legal Centre 2013 [65]	Greater London	N	Migrants No details	Not reported	Parents	Case studies. Methods of data collection and analysis are unclear	Vulnerably housed	Immigration status
Dexter 2016 [66]	London	N	Migrants Families seeking support under Section 17, as well as those who are already living on this support	“destitute migrant children, whose parents have no recourse to public funds”	*N* = 7 Children’s Society practitioners, *N* = 1 professional from Hackney Migrant Centre	Semi-structured interviews and a roundtable analysis of anonymised casefiles No analysis details	Varied, usually temporary	Poverty, immigration status
Jolly 2018 [67]	Birmingham	N	Migrants *N* = 15 immigrant families. Most from West Africa and Caribbean Households	24 children	Children	17 semi-structured interviews Qualitative: directive content analysis	Mainly temporary, or relocated	Immigration status, not in receipt of public funds.
Karim 2006 [68]	UK	N	General *N* = 35 families at follow-up.	Mean number of children = 3 (range 1 to 7).	Main carer, usually mother	Semi-structured interviews. Thematic content coding.	Hostel (or other temporary accommodation)	Domestic violence (20%), neighbour harassment (23%), relationship breakdown (23%) and eviction (17%)
Lawson 2015 [69]	Glasgow	Y	Gentrification 23 households, 21 of which ‘family households’ (≥1 adult + ≥1 child / young person)	Gentrification Not described	Parents	Longitudinal qualitative study (18-months). Semi structured interviews. Grounded theory	Being relocated due to regeneration	Regeneration (gentrification of local area).
Lawson 2016 [70]	Glasgow	Y	Gentrification 20 family households (10 at follow up)	Gentrification 40 children and young people	Parents	Longitudinal qualitative study (18-months). Semi structured interviews. Grounded theory	Being relocated due to regeneration	Regeneration (gentrification of local area).
Minton 2005 [71]	England and Scotland	Y	General “nearly 50 individuals”	Not reported	Children, parents, doctors, teachers, religious leaders, housing and homelessness professionals	Study design not reported. Analysis method unclear	Various, including homeless, in temporary accommodation, and precariously housed/moved round a lot.	Mainly eviction. Mostly poverty-related.
Moffatt 2016 [72]	North East England	N	General *N* = 38 tenants, all in receipt of welfare benefits	11 children altogether– 9 households had 1 child aged <18 years, 2 had 2, and 1 had 3 children.	Parents, service providers.	Semi-structured interviews Qualitative interpretive analysis	Living in social rented properties.	Poverty, bedroom tax.
Nettleton 2000 [73]	London	Y	General 20 families lived in London Boroughs	17 children (incl. siblings), age 7 to 18 years.	Children and their parents.	Qualitative Semi structured interviews. No reporting of analysis methods	Mortgage repossession (implies currently in rented accommodation)	Mortgage repossession
Office of the Deputy Prime Minister 2005 [74]	England	N	General *N* = 82 ethnic minority homeless households, 72 had a child, pregnancy, or children	No details	1 adult within each household interviewed, 73% female. Also: local authority service providers, charitable / voluntary sector service providers.	Interviews Thematic analysis,	Homeless	Various (DV, relationship breakdowns, family disputes, eviction, social exclusion, pregnancy, severe poverty, losing accommodation tied to a job, loss of National Asylum Support Service (NASS) accommodation, racial harassment.
Oldman 2000 [75]	UK	N	General 40 parents of children with physical disabilities or sensory impairments	Physical disabilities or sensory impairments	ParentsChildren	In depth interviews Qualitative analysis	Wide range of housing unsuitability and included those who had adapted or moved house in response to their housing needs.	Disabled child.
Price 2015 [76]	England & Wales	N	Migrants *N* = 91 interviewees, including parents, local authority workers, and 3^rd^ sector workers / advocates.	Not reported	Parents, local authority workers, advocates, voluntary sector staff.	Mixed methods – survey first, then in-depth interviews. No detail on analysis.	Various, usually temporary.	Poverty, immigration status, NRPF
Rowley 2020 [77]	UK	N	Migrants 9 adults; 5M, 4F Refugees	Not reported	Parents	Qualitative Interviews Thematic analysis	Homeless or temporarily housed	Refugee status
Thompson 2017 [78]	Newham, East London	Y	General 20 families (*n*=40) at wave 1, 15 families (*n*=28) at wave 2.	Age of children 11-16	Parents and children	Ethnography Described as narrative family interviews and Narrative analysis with Bakhtinian interpretation	Private renters, owned or were buying their own home.	Various including: overcrowding; joblessness; extremely poor quality of current housing; having ‘nowhere else to go’ (homelessness); and health problems
Tischler 2004 [79]	Leicester	Y	Domestic violence 49 homeless families (couple or single mother with children).	Families had a mean number of three children (range = 1–7).	Carer (usually mother)	Qualitative (semistructured) interviews Thematic analysis	Large statutory hostel for homeless parents and children	Domestic violence
Tischler 2007 [80]	Birmingham	Y	Domestic violence 28 homeless women with dependent children	Children aged ≥3 years. Median number of children = 2, range 1 to 6.	Mother	Semi-structured interviews Thematic analysis	Living in one of three local-authority-run hostels	Domestic violence
Tod 2015 [81]	Rotherham & Doncaster	N	General 35 families – low income households	Not reported	1 parent from each family and 25 health, education and social care staff	In-depth semi-structured individual and group interviews. Framework analysis	Mixture of privately owned, private rented and council rented	Low income households at risk of instability.
Warfa 2006 [82]	London	Y	Migrants Somali refugees in the UK (21 families)	School-age children.	Adults Professionals in supporting roles	In-depth group discussions	Refugees Frequent moves	Migration – Somali refugees
Watt 2018 [83]	East London	Y	General 5 young mothers (aged 18-24 years) and 12 female lone parents.	Not reported	Mothers	Interviews and participant observation	Hostel (homeless)	Family disputes, domestic violence and evictions
Wilcox 2000 [84]	Sheffield	N	Domestic violence 20 white working class women	Not reported	Mothers	In-depth interviews and participant observation. Analysis not reported	Council estate property	Fleeing domestic violence
Young Women’s Trust 2020 [85]	London	N	General Four young women living on low incomes	Not reported	Mothers	Focus group Analysis not reported	Unsuitable housing	Not reported
Children’s Commissioner 2017 [86]	England	N	General *N* = 15 children *N* = 25 parents and carers	No details	Children and parents	Observation “mosaic approach” No analysis details.	In rented accommodation	Poverty (worry about being evicted)
Children’s Commissioner 2019 [87]	England	Y	General Children and families living in temporary accommodation	No details	Children, parents, specialist health visitor team	Described only as: “visiting and speaking with participants, and conducting analysis”	In temporary accommodation, including B&Bs, converted office blocks and converted shipping containers.	Various, not clearly described.
Children’s Commissioner 2020 [88]	England, Scotland, Wales and Northern Ireland	N	General Described as “young people”	No details	Children	“Surveys, virtual visits to prisons, youth groups and children’s homes”. Analysis unclear	Unclear	Unclear – reasons include poverty and migration.
The Children’s Commissioner 2021 [89]	England	N	General *N* = 557,077 overall sample	Aged 4-17	Children	Online survey with focus groups and interviews Analysis unclear	Unclear	Unclear
Children’s Society 2017 [90]	England	N	Domestic violence Migrants *N* = 60 No details	Not reported	Children	Longitudinal fieldwork – annual semi-structured interviews. Thematic analysis	Temporary insecure housing	Various: to build a better life in the UK; to accommodate growing numbers of siblings; to live closer to extended family; parent with new partner; domestic violence, neighbourhood violence, family breakdown; eviction; poor quality housing; health problems; current accommodation temporary
Pinter 2020 (Children’s Society 2020) [91]	England	N	Migrants *N* = 11 parents / carers	Representing 21 children)	Parents / carers	Mixed methods – analysis of database and case notes, semi-structured interviews. No detail on analysis.	Temporarily housed, mainly	Immigration status and having No Recourse to Public Funds (NRPF)
Children’s Society 2020 [15]	UK	Y	General *N* = 24 participants recruited through schools	No details	Children	In-depth interviews, conducted annually over three years Thematic analysis	Various, mainly temporarily housed, or in ‘permanent’ or indefinite housing but with threat of moving.	Evicted for non-payment of rent, DV, being housed in temporary housing, unsuitability of housing
CPAG & CoE 2020 [92]	UK	N	General 21 parents (some lone parents / some part of couples) on low income	1-5 children	Parents	Interviews Thematic analysis	No details	Low income
CPAG 2020 [93]	UK	N	General *N*=129 professional informants	Not reported	117 social workers and 12 other professionals	Survey No details on analysis	Homeless	Low income
Hardy and Gillespie 2016 [94]	London	Y	General No details	No details	Parents	64 structured interviews (32 recorded). No details on analysis	Approached Newham Council to address a housing or homelessness need within the last year	Rent rises, cuts to benefits leading to rent arrears and family breakdown
Jones 2010 [95]	England	N	Domestic violence Adult and child sanctuary service users.	2 children, no details	Parents, children, professionals	Telephone interviews (semi structured). Thematic analysis	In own home	DV
Joshi 2015 [96]	England	N	General Family participation events: *N* = 16 parents; *N* = 15 children, Interviews: *N* = 9 parents	Children aged 0-4 years	Children, parents	Conversations “mosaic approach” Thematic analysis	Renting	Poverty, high rents
JRF 2017 [97]	England	Y	General 145 tenants experiencing forced moves and evictions Age 18+, 84 F, 61 M. 67 Families	Not reported	Parents	Qualitative interviews Thematic analysis	Facing forced move or eviction.	Facing a forced move or eviction, or who had experienced a forced move or eviction within the recent past.
JRF 2018 [98]	UK	N	General 72 participants in six case study areas	Not reported	Parents	Qualitative longitudinal panel study Analysis not reported	Home owners, private renters, social renters	Not defined.
JRF 2021 [99]	UK	N	General In poverty	Not reported	Insights from the JRF Grassroots Poverty Action Group (GPAG)	Charity annual report	Social housing	Not reported
Maternity Action 2022 [100]	England	N	Migrants *N* = 10 women with recent experience of pregnancy and asylum support	No details	Mothers	Online group discussion No analysis details	Temporary accommodation	Asylum seeking
Project 17 2018 [101]	London	N	Migrants *N* = 2 families	Children aged 6 to 12	Parents and children	“Informal qualitative research”	Homeless	Refusal of Section 17 support (for migrant children or children of adult migrants with no recourse to public funds)
Project 17 2019 [102]	London	N	Migrants 11 families being supported under Section 17.	*N* = 17 children aged 7-17	Children	Mixed methods approach. No analysis details	Temporary, transient, some were street homeless for periods of time.	Immigration status, no recourse to public funds
RCPCH 2017a [103]	London	Y	General No details	No details	Parents, carers and young people	Workshop No analysis details	Living in temporary accommodation	Poverty
RCPCH 2017b [41]	London	N	General *N*=266 professionals	No details	Professionals	Survey No analysis details	Living in poverty	Not reported
Renter's Reform Coalition 2022 [104]	UK	Y	General No details	Not reported	Parents	Not reported	Private renters	Eviction and increasing costs.
Scottish Women’s Aid 2015 [105]	Fife	Y	Domestic violence *N* = 4 (interviews), women who had experienced or been at risk of homelessness as a result of domestic abuse.	3 had dependent children,	Parents	Participatory action research Mixed methods survey/interviews No analysis details	Homeless / TA	Domestic abuse
Shelter 2004b [106]	UK	Y	General Homeless children	*N*=29 children 17M, 12F Age 4-16.	Children	Writing and drawing in activity books, completing a questionnaire and participating in drama exercises. Follow up interviews No analysis details	All of the children were, or had recently been, homeless. Rehoused in private/social rented or hostels	Relationship breakdown or eviction, or the need to escape violence or racist abuse.
Shelter 2004d [107]	England	N	Domestic violence No details	Not reported	Parents	Not reported	Temporary accommodation	Fleeing domestic violence
Shelter 2012 [108]	England	Y	General No details	Not reported	Parents	Policy briefing. No analysis methods reported.	Private rental	Private rental insecurity
Shelter 2014 [109]	UK	Y	General 171 adults. 71 women and 57 men at 19 months, “with a fairly even split of single households and households with children".	No details on children.	Parents	Qualitative semi-structured and unstructured interviews. 19 month follow up. No analysis details	Homeless - recently been resettled into private rented accommodation	Not stated
Shelter 2004c [110]	England	Y	General 194 families: 72% lone parents, 28% couples.	62% had a child/children under the age of four living with them, 38% had a child/children aged 5-10 yrs living with them, 26% had a child/children aged 11-16 living with them, 9% had a child/children aged 17-18 living with them.	Parents	Questionnaires. In-depth case history interviews. No analysis details	Temporary accommodation	Not reported
Shelter 2015 [111]	England	Y	General 20 families 6 teachers/ learning mentors	14 families had children under 10 years.	Parents/teachers	Qualitative interviews. Thematic framework analysis.	Families in non-self-contained accommodation, such as B&Bs and hostels	Not reported
Shelter 2016b [112]	UK	Y	General 25 parents living in emergency accommodation	Not reported	Parents	Qualitative interviews. Thematic framework analysis.	Living in emergency accommodation (some for 6 months or more)	Not clear
Shelter 2016c [113]	UK	N	General *N*=19, including 11 families with dependent children, three couples and four single people	11 families with dependent children (no details)	Parents	In depth interviews. Qualitative. Thematic analysis.	Currently, or have previously been at risk of becoming homeless.	Debt
Shelter 2017a [114]	England	Y	General 23 families currently living in emergency accommodation, or who had left within the last three months	10 children aged 6-16 years.	Parents and children	Qualitative interviews. Thematic framework analysis.	Emergency accommodation	Not reported
Shelter 2017b [115]	England	Y	General Primary and secondary schools populations	No details	8 teachers and 3 education Professionals. 10 different primary and secondary schools	Qualitative Interviews. Thematic analysis	Homeless	Not reported
Shelter 2018 [116]	UK	N	General Social housing tenants and private rented (no details on individual children)	Not reported		Mixed methods study. Qualitative data presented as case studies No analysis details	Social housing tenants plus struggling private renters	Varied – most at risk rather than homeless.
Shelter 2021 [117]	UK	N	General No details	Not reported	Professionals (no details)	Website with case study quotations	Evicted from private rented accommodation	Eviction
White 2008 [118]	England	N	General 9 family case studies (with 18 families, 2 per case study), based on 9 (9-day) site visits.	No details	Families (any family member aged ≥5 years), FIP staff, local agencies and services that work with a FIP.	Mixed methods evaluation: 9 case studies, 44 telephone interviews. No analysis details	Housed, mostly local authority renting, most families had housing enforcement actions (threat of removing tenants).	Anti-social behaviour

We identified four distinct populations for which research evidence was available during the process of study selection and data extraction:

- General population (evidence relating to housing insecurity in general) (reported in 40 papers);
- Domestic violence population (children and young people experiencing housing insecurity associated with domestic violence) (reported in nine papers);
- Migrant, refugee and asylum seeker population (children and young people experiencing housing insecurity associated with migration status) (reported in 13 papers);
- Relocation population (evidence relating to families forced to relocate due to planned demolition) (reported in two papers).

Evidence relating to each of these populations was synthesised separately as the specific housing circumstances may impact health and wellbeing differently and we anticipated that specific considerations would relate to each population. Some studies reported evidence for more than one population.

### Quality of evidence

The quality of evidence varied across the studies, with published literature generally being of higher quality than grey literature and containing more transparent reporting of methods, although reporting of methods of data collection and analysis varied considerably within the grey literature. All 18 peer-reviewed studies reported an appropriate methodology, addressing the aim of the study with an adequate design. Eleven of the 18 peer-reviewed studies reported ethical considerations, and only two reported reflexivity. Most studies had an overall assessment of moderate-high quality (based on the endorsement of most checklist items) and no studies were excluded based on quality. Most of the grey literature originated from known and valued sources (e.g., high-profile charities specialising in poverty and housing, with the research conducted by university-based research teams). Although methodologies and methods were often poorly described (or not at all), primary data in the form of quotations was usually available and suitable to contribute to the development of themes within the evidence base as a whole. Quality appraisals of included studies are presented in Supplementary Tables 3 and 4, Additional File 4.

### Housing insecurity and the health and wellbeing of children and young people

**Fig. 3:**
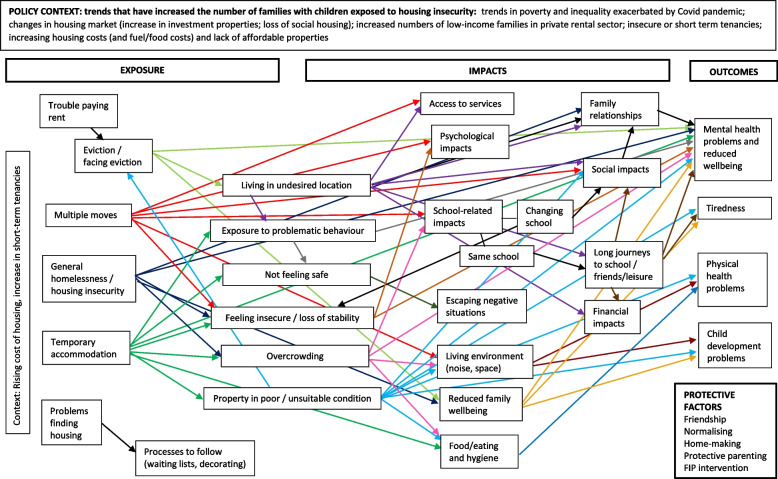
Conceptual framework for the relationship between housing insecurity and health and wellbeing in the general population

**Fig. 4:**
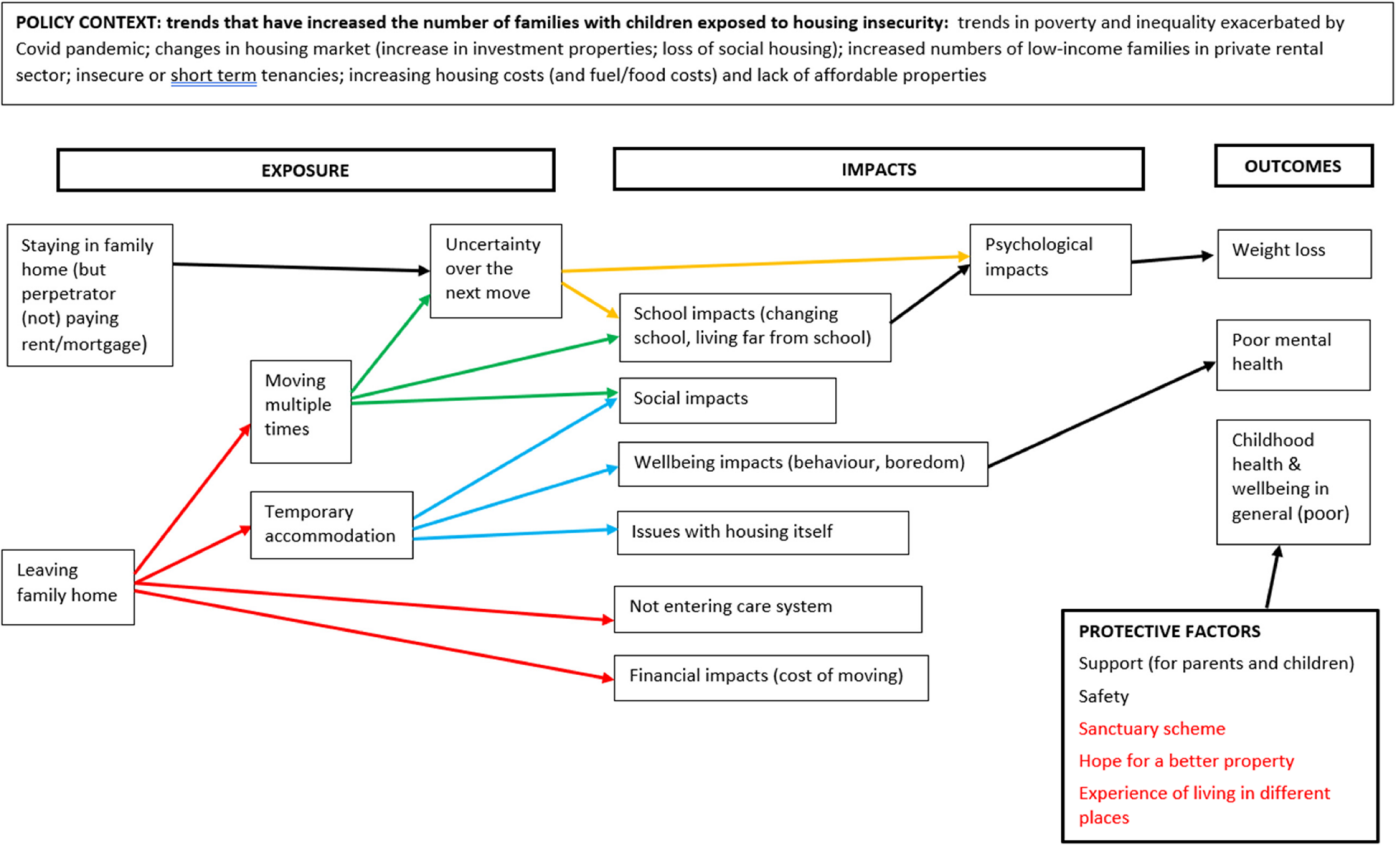
Conceptual framework for the relationship between housing insecurity and health and wellbeing in the domestic violence population

**Fig. 5:**
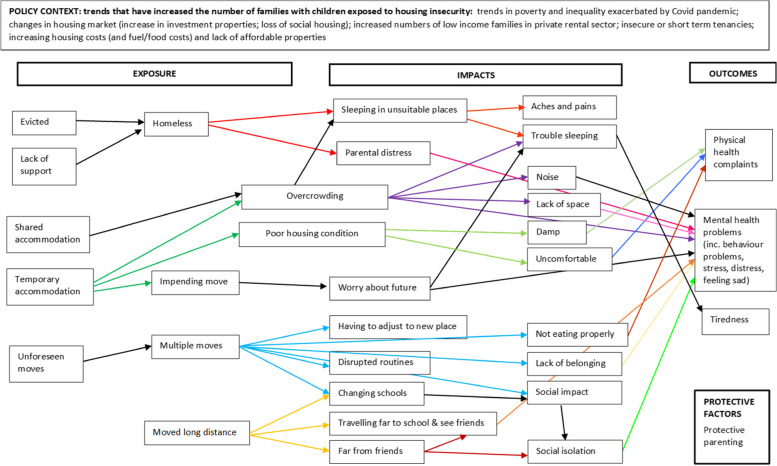
Conceptual framework for the relationship between housing insecurity and health and wellbeing in the migrant, refugee and asylum seeker population

**Fig. 6:**
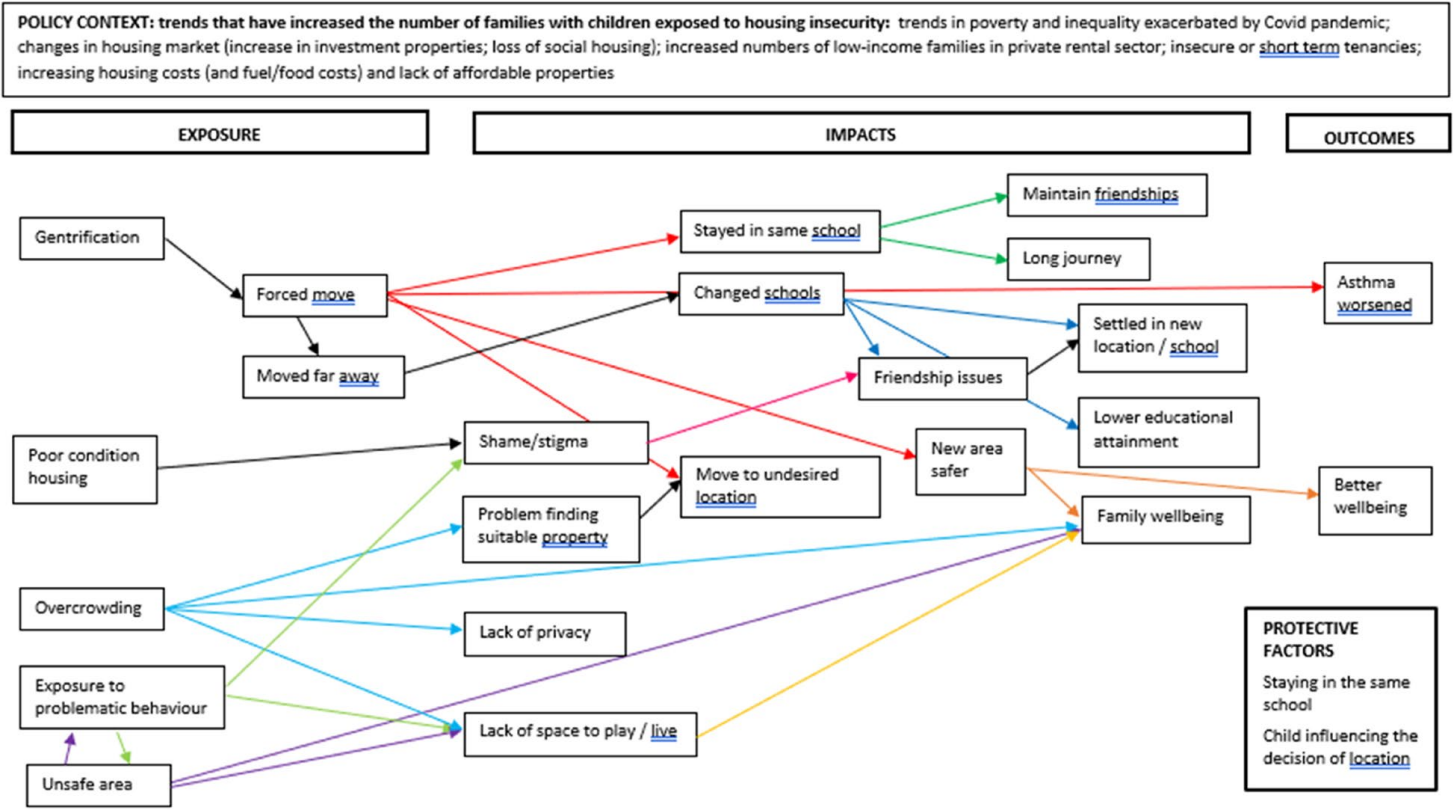
Conceptual framework for the relationship between housing insecurity and health and wellbeing in the relocation population

The updated conceptual framework for the impact of housing insecurity on the health and wellbeing of children aged 0–16 years in family units is presented in Fig. 3 for the general population, Fig. 4 for the domestic violence population, Fig. 5 for the refugee/migrant/asylum seeker population, and Fig. 6 for the relocation population (arrows represent links identified in the evidence and coloured arrows are used to distinguish links relating to each element of the model). Table 3 outlines the themes, framework components and studies reporting data for each theme.

**Table 3:** Evidence relating to each theme and framework component

**Theme**	**Part of conceptual framework**	**Paper / report**
Trouble paying rent	Exposure	Child Poverty Action Group and Church of England 2020 [92] Children’s Society 2020 [15] Clarke 2017 [97] Shelter 2014 [109] Shelter 2018 [116]
Eviction/facing eviction	Exposure	Children’s Society 2017 [90] Children’s Society 2020 [15] Shelter 2014 [109] Shelter 2018 [116] Project 17 2019 [102]
Lack of support from local authority with housing	Exposure	Project 17 2018 [101]
Unforeseen moves	Exposure	Warfa 2006 [82] Rowley 2020 [77]
Multiple moves	Exposure	Children’s Society 2020 [15] Minton 2005 [71] Rowley 2020 [77] Shelter 2014 [109] Shelter 2018 [116] Warfa 2006 [82]
General homelessness/housing insecurity	Exposure	Children’s Commissioner for England 2019 [87] Children’s Society 2017 [90] Children’s Society 2020 [15]
Temporary accommodation	Exposure	Children’s Commissioner 2020 [88] Children’s Commissioner for England 2019 [87] Children’s Society 2017 [90] Children’s Society 2020 [15] Children’s Society 2020 [91] Croucher 2018 [98] Dexter 2016 [66] Hardy 2016 [94] Jolly 2018 [67] Minton 2005 [71] Office of the Deputy Prime Minister 2005 [74] Price 2015 [76] Project 17 2019 [102] Renters’ Reform Coalition 2022 [104] Rowley 2020 [77] Royal College of Paediatrics and Child Health 2017 [41] Shelter 2004 [106] Shelter 2014 [109] Shelter 2015 [111] Shelter 2016 [112] Shelter 2016 [113] Shelter 2017 [114] Shelter 2017 [115] Shelter 2018 [116] Tod 2016 [81]
Problems finding housing	Exposure	Children’s Society 2020 [15] Clarke 2017 [97] Croucher 2018 [98] Shelter 2018 [116] Shelter 2021 [117] Young Women’s Trust 2020 [85]
Domestic violence – staying in the family home	Exposure	Watt 2018 [83]
Domestic violence – leaving the family home	Exposure	Children’s Society 2017 [90] Scottish Women's Aid 2015 [105]
Forced move due to gentrification-related demolition	Exposure	Lawson 2015 [69] Lawson 2016 [70]
Living in undesired location	Exposure/impact	Children’s Society 2020 [15] Children’s Commissioner for England 2019 [87] Clarke 2017 [97] Renters’ Reform Coalition 2022 [104] Shelter 2014 [109] Watt 2018 [83]
Exposure to problematic behaviour	Exposure/impact	Children’s Commissioner for England 2019 [87] Children’s Society 2017 [90] Children’s Society 2020 [15] Clarke 2017 [97] Lawson 2015 [69] Shelter 2004 [106] Shelter 2004 [107] Shelter 2015 [111] Shelter 2016 [112] Shelter 2017 [114] Watt 2018 [83]
Not feeling safe	Exposure/impact	Children’s Commissioner for England 2019 [87] Children’s Society 2017 [90] Children’s Society 2020 [15] Clarke 2017 [97] Lawson 2015 [69] Royal College of Paediatrics and Child Health 2017 [103] Shelter 2004 [107] Shelter 2014 [109] Shelter 2016 [112] Shelter 2016 [113] Shelter 2017 [114]
Feeling insecure/loss of stability	Exposure/impact	Children’s Commissioner for England 2019 [87] Children’s Society 2017 [90] Children’s Society 2020 [15] Shelter 2014 [109] Shelter 2015 [111] Shelter 2016 [113] Shelter 2017 [114] Shelter 2017 [115] Shelter 2018 [116] Young Women’s Trust 2020 [85]
Overcrowding	Exposure/impact	Bradley 2020 [64] Children’s Society 2017 [90] Children’s Society 2020 [15] Children’s Society 2020 [91] Clarke 2017 [97] Croucher 2018 [98] Hardy 2016 [94] Jolly 2018 [67] Lawson 2016 [70] Maternity Action 2022 [100] Minton 2005 [71] Moffatt 2017 [72] Project 17 2019 [102] Royal College of Paediatrics and Child Health 2017 [41] Royal College of Paediatrics and Child Health 2017 [103] Shelter 2004 [110] Shelter 2014 [109] Shelter 2015 [111] Shelter 2016 [112] Shelter 2016 [113] Shelter 2017 [114] Shelter 2018 [116] Thompson 2017 [78] Tischler 2004 [79] Watt 2018 [83]
Property in poor/unsuitable condition	Exposure/impact	Bowyer 2015 [63] Child Poverty Action Group and Church of England 2020 [92] Children’s Society 2017 [90] Children’s Society 2020 [91] Children’s Commissioner 2020 [88] Children’s Commissioner for England 2017 [86] Children’s Commissioner for England 2019 [87] Child Poverty Action Group 2020 [93] Clarke 2017 [97] Croucher 2018 [98] Jolly 2018 [67] Joshi 2015 [96] Minton 2005 [71] Office of the Deputy Prime Minister 2005 [74] Oldman 2000 [75] Price 2015 [76] Project 17 2019 [102] Renters’ Reform Coalition 2022 [104] Rowley 2020 [77] Royal College of Paediatrics and Child Health 2017 [41] Shelter 2004 [106] Shelter 2014 [109] Shelter 2016 [112] Shelter 2018 [116] Thompson 2017 [78] Tischler 2000 [80] Tod 2016 [81] Watt 2018 [83]
Poor access to services	Impact	Child Poverty Action Group 2020 [93] Children’s Society 2017 [90] Coram Children's Legal Centre 2013 [65] Minton 2005 [71] Royal College of Paediatrics and Child Health 2017 [41] Warfa 2006 [82] Watt 2018 [83] Young Women’s Trust 2020 [85]
Psychological impacts	Impact	Children’s Society 2020 [15] Shelter 2017 [115]
School-related impacts	Impact	Child Poverty Action Group 2020 [93] Children’s Commissioner 2020 [88] Children’s Society 2017 [90] Children’s Society 2020 [15] Children’s Society 2020 [91] Coram Children's Legal Centre 2013 [65] Dexter 2016 [66] Hardy 2016 [94] Lawson 2015 [69] Lawson 2016 [70] Minton 2005 [71] Project 17 2019 [102] Rowley 2020 [77] Royal College of Paediatrics and Child Health 2017 [41] Scottish Women's Aid 2015 [105] Shelter 2004 [106] Shelter 2004 [107] Shelter 2012 [108] Shelter 2014 [109] Shelter 2015 [111] Shelter 2016 [112] Shelter 2016 [113] Shelter 2017 [114] Shelter 2017 [115] Shelter 2018 [116]
Escaping negative situations	Impact	Children’s Society 2017 [90] Children’s Society 2020 [15]
Living environment (noise/space)	Impact	Bowyer 2015 [63] Children’s Commissioner for England 2019 [87] Children’s Society 2020 [15] Children’s Society 2020 [91] Lawson 2016 [70] Project 17 2019 [102] Royal College of Paediatrics and Child Health 2017 [103] Shelter 2004 [110] Shelter 2004 [106] Shelter 2014 [109] Shelter 2016 [112] Tischler 2000 [80]
Reduced family wellbeing	Impact	Child Poverty Action Group 2020 [93] Children’s Society 2020 [15] Coram Children's Legal Centre 2013 [65] Project 17 2019 [102] Royal College of Paediatrics and Child Health 2017 [41] Shelter 2004 [106] Shelter 2014 [109] Shelter 2015 [111]
Food/eating and hygiene	Impact	Children’s Commissioner 2020 [88] Children’s Commissioner for England 2019 [87] Children’s Society 2020 [15] Children’s Society 2020 [91] Jolly 2018 [67] Joseph Rowntree Foundation 2021 [99] Minton 2005 [71] Office of the Deputy Prime Minister 2005 [74] Price 2015 [76] Project 17 2019 [102] Renters’ Reform Coalition 2022 [104] Rowley 2020 [77] Royal College of Paediatrics and Child Health 2017 [41] Royal College of Paediatrics and Child Health 2017 [103] Shelter 2004 [106] Shelter 2014 [109] Shelter 2016 [112] Shelter 2016 [113] Shelter 2017 [115] Shelter 2018 [116] Tod 2016 [81]
Family relationships	Impact	Children’s Society 2020 [15] Shelter 2004 [106] Warfa 2006 [82]
Social impacts	Impact	Bowyer 2015 [63] Children’s Society 2017 [90] Children’s Society 2020 [15] Nettleton 2000 [73] Project 17 2019 [102] Shelter 2004 [106] Shelter 2015 [111] Shelter 2016 [112] Shelter 2017 [114] Shelter 2017 [115] Shelter 2018 [116] Thompson 2017 [78]
Long journeys	Impact	Children’s Commissioner 2020 [88] Children’s Commissioner for England 2019 [87] Children’s Society 2017 [90] Children’s Society 2020 [15] Dexter 2016 [66] Hardy 2016 [94] Lawson 2016 [70] Project 17 2019 [102] Rowley 2020 [77] Scottish Women's Aid 2015 [105] Shelter 2004 [106] Shelter 2015 [111] Shelter 2016 [112] Shelter 2016 [113] Shelter 2017 [114]
Financial impacts	Impact	Children’s Commissioner for England 2019 [87] Children’s Society 2020 [15] Child Poverty Action Group 2020 [93] Croucher 2018 [98] Hardy 2016 [94] Renters’ Reform Coalition 2022 [104] Scottish Women's Aid 2015 [105] Shelter 2016 [112] Shelter 2017 [114] Wilcox 2000 [84]
Not entering the care system	Impact	Children’s Society 2017 [90]
Sleeping in unsuitable places	Impact	Project 17 2019 [102]
Adjusting to a new area with language barriers	Impact	Warfa 2006 [82]
Moving to a safer area and/or better property	Impact	Children’s Society 2020 [15] Lawson 2015 [69]
Mental health problems and reduced wellbeing	Outcome	Bowyer 2015 [63] Children’s Commissioner for England 2019 [87] Children’s Commissioner for England 2021 [89] Children’s Society 2017 [90] Children’s Society 2020 [15] Clarke 2017 [97] Dexter 2016 [66] Jones 2010 [95] Joshi 2015 [96] Karim 2006 [68] Lawson 2015 [69] Minton 2005 [71] Nettleton 2000 [73] Royal College of Paediatrics and Child Health 2017 [41] Royal College of Paediatrics and Child Health 2017 [103] Shelter 2004 [110] Shelter 2004 [106] Shelter 2004 [107] Shelter 2014 [109] Shelter 2015 [111] Shelter 2016 [112] Shelter 2016 [113] Shelter 2017 [114] Shelter 2017 [115] Tischler 2004 [79] Warfa 2006 [82] White 2008 [118] Wilcox 2000 [84]
Tiredness	Outcome	Children’s Commissioner for England 2019 [87] Children’s Society 2020 [15] Dexter 2016 [66] Project 17 2019 [102] Rowley 2020 [77] Royal College of Paediatrics and Child Health 2017 [41] Shelter 2014 [109] Shelter 2015 [111] Shelter 2016 [112] Shelter 2017 [114] Shelter 2017 [115]
Physical health problems	Outcome	Children’s Commissioner for England 2017 [86] Children’s Society 2017 [90] Children’s Society 2020 [91] Lawson 2015 [69] Maternity Action 2022 [100] Minton 2005 [71] Oldman 2000 [75] Project 17 2019 [102] Royal College of Paediatrics and Child Health 2017 [41] Shelter 2014 [109] Shelter 2015 [111] Shelter 2016 [112] Shelter 2016 [113] Shelter 2017 [115] Thompson 2017 [78] Tischler 2004 [79] Tod 2016 [81] Watt 2018 [83]
Child development problems	Outcome	Children’s Commissioner for England 2019 [87] Shelter 2015 [111] Shelter 2016 [113]
Weight loss	Outcome	Watt 2018 [83]
Friendship	Protective factors	Children’s Society 2017 [90] Children’s Society 2020 [15] Shelter 2017 [114]
Keeping the same school	Protective factors	Children’s Society 2017 [90] Children’s Society 2020 [15] Lawson 2016 [70] Shelter 2012 [108]
Normalising	Protective factors	Children’s Society 2017 [90] Children’s Society 2020 [15] Backett-Milburn 2003 [62]
Home-making	Protective factors	Children’s Society 2020 [15]
Protective parenting	Protective factors	Children’s Society 2017 [90] Children’s Society 2020 [91] Lawson 2016 [70] Shelter 2016 [113]
Interventions (FIP, peer-led parenting programme)	Protective factors	Bradley 2020 [64] White 2008 [118]
Specialist support for children who have experienced domestic violence	Protective factors	Bowyer 2015 [63] Tischler 2000 [80] Tischler 2004 [79]
Safety (including the Sanctuary Scheme)	Protective factors	Jones 2010 [95]

### Exposure

Exposures are conceptualised as the manifestations of housing insecurity – that is, how the children and young people experience it – and housing insecurity was experienced in multiple and various ways. These included trouble paying for housing, eviction or the prospect of eviction, making multiple moves, living in temporary accommodation, and the inaccessibility of suitable accommodation.

Fundamentally, a key driver of housing insecurity is poverty. Parents and, in some cases, young people cited the high cost of housing, in particular housing benefit not fully covering the rent amount [116], trouble making housing payments and falling into arrears [15, 92, 97]. Sometimes, families were evicted for non-payment [15, 102], often linked to the rising cost of housing [109] or loss of income [102]. Some children and young people were not aware of reasons for eviction [90], and the prospect of facing eviction was also a source of housing insecurity [116].

The cost of housing could lead to families having to move multiple times [116], with lack of affordability and the use of short-term tenancies requiring multiple moves [109, 116]. Children and young people were not always aware of the reasons for multiple moves [15]. Multiple moves could impact upon education and friendships [77, 82].

Living in temporary housing was a common experience of housing insecurity [15, 71, 87, 90, 94, 98, 111–114]. Temporary housing caused worry at the thought of having to move away from school and friends [91] and acute distress, which manifested as bedwetting, night waking and emotional and behavioural issues at school [66]. Living in a hostel for a period of time could lead to friendship issues due to not being able to engage in sleepovers with friends [102].

The inaccessibility of suitable accommodation also contributed to insecurity. Sometimes, when a family needed to move, they had to fulfil certain requirements, for instance, to decorate their overcrowded 3-bedroom accommodation to be eligible for a more suitable property [15]. Further, some families encountered the barrier of landlords who would not accept people on benefits [15, 85, 117]. Waiting lists for social housing could be prohibitively long [97, 98, 116].

### Dual exposures and impacts

Some phenomena were found to be both exposures and impacts of housing insecurity, in that some issues and experiences that were impacts of housing insecurity further exacerbated the living situation, causing further insecurity. These included not feeling safe, exposure to problematic behaviour, living far away from daily activities, overcrowding, and poor or unsuitable condition properties.

Not feeling safe was frequently reported by children and young people, and by parents in relation to the safety of children and young people. Parents and children and young people described being moved to neighbourhoods or localities [15, 69, 87, 90, 103] and accommodation [87, 97, 109, 112–114] that did not feel safe. For one family, this was due to racial abuse experienced by a parent while walking to school [69]. In one case, a young person’s perception of safety improved over time, and they grew to like the neighbours and area [15], although this was a rare occurrence.

Often, this experience of being unsafe was due to exposure to problematic behaviour in or around their accommodation, including hearing other children being treated badly [112], being exposed to violence (including against their parents) [111, 112, 114], witnessing people drinking and taking drugs [69, 83, 90, 111, 112, 114], finding drug paraphernalia in communal areas [112, 114] or outside spaces [69], hearing threats of violence [111], hearing shouting and screaming in other rooms [114], witnessing people breaking into their room [83], and witnessing their parent/s receiving racist abuse and being sworn at [83].

*‘There’s a lot [of] drugs and I don’t want my kids seeing that… One time he said ‘mummy I heard a woman on the phone saying ‘I’m going to set fire to your face’’ She was saying these things and my son was hearing it.’ (*[111]*, p.15)*

Another impact related to the family and children and young people being isolated and far away from family, friends, other support networks, work, shops, school and leisure pursuits due to the location of the new or temporary housing [15, 83, 87, 97, 104, 109]. This affected education, friendships, finances and access to services (see ‘Impacts’).

Overcrowding was another issue that was both a source or feature of housing insecurity, as this created a need to move, as well as being an impact, in that families moved to unsuitable properties because they had little alternative. Overcrowding was largely a feature of temporary accommodation that was too small for the family [67, 91], including hostels/shared houses where whole families inhabited one room and washing facilities were shared [100, 102]. In turn, overcrowding could mean siblings sharing a room and/or bed [15, 41, 64, 71, 78, 109, 111–114, 116] (which could lead to disturbed sleep [15]), children/young people or family members sleeping on the floor or sofa [15, 71, 102, 110] (which caused aches and pains in children/young people; [100]), children/young people sharing a room with parents [64, 71, 94, 109, 111–114], a room being too small to carry out day to day tasks [112–114], a lack of privacy in general (e.g., having to change clothes in front of each other) [70, 111, 112, 114], living in close proximity to other families [114], and cramped conditions with little room to move when too many people and possessions had to share a small space [15, 64, 90, 97, 103, 109, 114].

*It’s all of us in one room, you can imagine the tension…. everyone’s snapping because they don’t have their own personal space …it’s just a room with two beds. My little brother has to do his homework on the floor.’ (*[97]*, p..43)*

It was thus difficult for children and young people to have their own space, even for a short time [98], including space to do schoolwork [102, 103], play [91] or invite friends over [103]. Families sometimes ended up overcrowded due to cohabiting with extended family [110] or friends [91, 102] (‘hidden homelessness’). Other families outgrew their property, or anticipated they would in future, when children grew older [70, 116]. Overcrowding sometimes meant multiple families inhabiting a single building (e.g., a hostel or shelter), where single parents had difficulties using shared facilities, due to not wanting to leave young children alone [100]. Overcrowding could also lead to children feeling unsafe, including being scared of other people in shared accommodation [102], experiencing noise [102], and feeling different from peers (due to not having their own room or even bed) [102]. Living in overcrowded conditions could lead to, or exacerbate, boredom, aggressive behaviour, and mental health problems among children and young people (see ‘Outcomes’) [72, 79, 91]. Overcrowded conditions caused a ‘relentless daily struggle’ for families ([83], p.48).

Similarly, the need to take whatever property was on offer led to families living in properties in poor condition, which in turn could exacerbate housing insecurity, both because families needed to escape the poor condition housing and because they were reluctant to complain and ask for repairs on their current property in case the landlord increased the rent or evicted them [86, 96]. Eviction was perceived as a real threat and families described being evicted after requesting environmental health issues [74] and health and safety issues [116] be addressed. Families experienced issues relating to poor condition properties, including accommodation being in a poor state of decoration [98], broken or barely useable fixtures and fittings [86, 90, 96], no laundry or cooking facilities [102], no electricity [67], no or little furniture [67, 102], broken appliances [71, 96, 97], structural failings [97], unsafe gardens [90], mould [71, 90, 96, 97, 104, 109], and bedbugs and/or vermin [67, 76, 77]. Even where the property condition was acceptable, accommodation could be unsuitable in other ways. Many families with young children found themselves living in upper floor flats, having to navigate stairs with pushchairs and small children [71, 74, 78, 83, 87, 92, 109]. One study reported how a family with a child who had cerebral palsy and asthma were refused essential central heating and so had to request a property transfer [75]. Lack of space to play was a particular issue in relation to temporary accommodation, often due to overly small accommodation or a vermin infestation [80, 87, 91]. In small children, the effects included health and safety risks [87, 112] and challenges keeping them occupied [112]. In older children and young people, a lack of space meant a lack of privacy [63, 112]. School holidays could be particularly challenging, particularly when outside play spaces were unsuitable due to safety concerns (e.g., people selling drugs, broken glass) [87, 106], and some temporary accommodation restricted access during the daytime [112]. With shared temporary accommodation, such as a refuge or hostel, came the threat of possessions being removed by others [80].

### Impacts

Impacts are defined here as intermediate outcomes that may mediate the effects of housing insecurity on health and wellbeing, for instance, the psychological, social, and environmental consequences of experiencing housing insecurity. According to the evidence reviewed, these were overwhelmingly negative, with only a very small number of positive impacts, and, in many cases, these were offset by other negative impacts. Impacts on friendships, education, family relationships, diet, hygiene, access to services, feelings of being different, feelings of insecurity, parental wellbeing, the financial situation of the family, experiences of noise, leaving negative situations behind, and other impacts, such as leaving pets behind and time costs, were noted. Overlaying all of the above was a lack of choice and control experienced by the children/young people and their families.

A particularly large and disruptive impact of housing insecurity was the effect on friendships and social networks. Over multiple moves, children and young people faced the challenge of building new social networks and reputations each time [15, 90, 106], and worried about maintaining existing friendships [90]. The beneficial side to this was the potential to have friends all over town, although this was offset by difficulty in forming close friendships due to frequent moves [15]. Children and young people in temporary, overcrowded or poor condition accommodation often felt ashamed of their housing and concealed it from their friends [15, 73, 78, 111, 112, 114, 115], and in one case missing out on sleepovers with friends [102]. Moving far from friends presented difficulties in maintaining friendships and a social life, leading to boredom and isolation [102, 114]. The threat of an impending long-distance move could cause sadness and worry [114] and young people missed the friends they had left behind [15, 90]. Other associated social impacts of housing insecurity exacerbated by the wider experience of poverty included turning turn down invitations to go out with friends for financial reasons [115] or to avoid leaving a parent alone with younger sibling/s [114], and feeling different from peers, either because of looking unkempt or lacking in confidence [115].

Another key impact of housing insecurity was the effect on education, and this was closely intertwined with friendship impacts. Faced with moving, often multiple times, sometimes to uncertain locations, families were faced with the decision to keep the same school or to change schools. Multiple moves and/or an unfeasibly long journey to school, led to either a decision to, or anticipating the prospect of having to, change schools [15, 66, 90, 91, 102, 106, 108, 111, 116]. This could in turn impact on the child’s sense of stability, academic performance and friendships [90, 105, 106, 111, 115, 116] and make them feel sad [102]. In the case of one family, staying at the same school during a move resulted in decreased educational attainment [69].

Staying at the same school created some stability and allowed for friendships and connections with teachers and the school to be maintained [15, 102]. This was, however, quite often the only option, due to the family not knowing their next location, and thus which school they would be near [15, 102, 113], and was not without issues. Those who were unhappy with school were thus effectively prevented from changing schools due to housing insecurity [15, 90]. Families were often re-housed at a considerable distance from the school [15, 70, 93, 94, 113]. This meant having to get up very early for a long journey by public transport [15, 66, 70, 77, 88, 90, 94, 102, 105, 106, 111, 113], which also caused problems maintaining friendships [115], increased tiredness and stress [15, 66, 77, 102, 111, 113–115] and left little time for homework and extra-curricular activities [113–115]. Some children and young people stayed with friends or relatives closer to school on school nights, although these arrangements were not sustainable longer-term [15, 90].

Living in temporary housing was associated with practical challenges in relation to schooling, for instance, keeping track of uniform and other possessions, limited laundry facilities, and limited washing facilities [112, 115]. Parents noted academic performance worsened following the onset of housing problems [111, 113, 116]. Limited space and time to do homework or revision [111–115], tiredness and poor sleep [111, 113], travelling and disrupted routines [114], disruptions from other families (e.g. in a hostel) [114], a lack of internet connection [114], and the general impact of the housing disruption [111, 113, 116] made it challenging for those experiencing housing insecurity to do well at school. Families often had to wake up early to access shared facilities in emergency accommodation before school [113, 114]. Some children and young people missed school altogether during periods of transience, due to multiple moves rendering attendance unviable [71, 106, 111], lack of a school place in the area [109], or not being able to afford transport and lunch money [81], which in turn affected academic performance [106, 111].

*‘Their education was put on hold. My daughter was ahead on everything in her class and she just went behind during those two weeks.’ *([111]*, p.15)*

Children and young people also experienced an impact on immediate family relationships. Housing insecurity led to reduced family wellbeing [82], and family relationships becoming more strained, for instance, due to spending more time at friends’ houses that were far away [15]. In some cases, however, housing insecurity led to improved family relationships, for instance, in terms of a non-resident father becoming more involved [15], or children feeling closer to their parents [106].

Some impacts related to the child’s health and wellbeing. Impacts on diet were reported, including refusal of solid food (which affected growth) [113], stress and repeated moves leading to not eating properly (which resulted in underweight) [91], insufficient money to eat properly [15, 99, 106], a lack of food storage and preparation space [102, 103, 112], and a hazardous food preparation environment [112]. Unsuitable temporary accommodation, including converted shipping containers, hostels, B&Bs and poorly maintained houses were particularly likely to be associated with a wide range of other well-being related impacts. Unsuitable accommodation presented various problems, including excessive heat, dripping water, overcrowding, damp, dirt, electrical hazards, vermin, flooding and a lack of washing and laundry facilities [41, 67, 71, 74, 76, 77, 81, 87, 88, 102, 104, 106, 109, 112, 116]. Moving could also impact on access to services and continuity of care, including being unable to register with general practitioners [82], and difficulty in maintaining continuity of medical care [65].

Psychological impacts of housing insecurity included feeling different from peers [115], feeling disappointed in each new property after being initially hopeful [15], and having trouble fitting in, in a new area [15]. Feeling insecure (including uncertainty over when and where the next move will be, or if another move is happening) was a further impact of living in insecure housing situations (including temporary housing, making multiple moves, being evicted) [15, 87, 90, 114, 116], leading to stress and worry [15, 114].

*One of the major issues that [she] says affects her mental health is the uncertainty of their situation. She says it is hard to not know where they will be staying one night to the next. It is also difficult to adjust to living without her furniture and clothes (*[114]*, p.17)*

Multiple moves, or anticipating a move, disrupted children and young people’s sense of continuity and led to the experience of a loss of security and stability more generally [15, 85, 87]. This led children and young people to feel responsible for helping and providing support to their parents, including hiding their feelings [111, 114], or not requesting things be bought [15, 113]. Children and young people also felt a sense of displacement and a lack of belonging [15, 115]. Loss of stability and security triggered a desire for stability, to be able to settle, have friends over, and not have to worry about moving [109].

Housing insecurity also had a negative effect on parent-wellbeing, and this impacted the wellbeing of young people both directly [15, 65, 102, 106] and indirectly through increased arguments and family stress [15, 93] and reduced parental ability to care for children with chronic conditions [41]. Parents also perceived their reduced wellbeing as negatively impacting their children's development [41]. The threat of sanctions for missed housing payment could lead to reduced well-being among the whole family, characterised by feelings of despair, failure and a loss of hope [93].

Moving also had a financial impact on families. Moving into much smaller temporary accommodation meant that possessions had to be left behind, with the family having to pay for decorating, carpets, curtains and furniture each time they moved [15, 84, 98, 104, 105], incurring considerable debt [98]. If the new location was far away from school, family, friends and, in some cases, shops, then the family incurred travel costs [15, 87, 94, 112, 114]. Because of all this, children and young people’s requests for possessions or experiences (e.g., trips out) were refused [113].

Excessive noise was another disruption that children and young people experienced in connection with their precarious housing situation. Sources of noise were traffic on a main road [15] a factory nearby [110], or from other people in a B&B, hotel, hostel, or neighbouring properties [15, 91, 102, 106, 112], and could disrupt sleep and daily activities.

If their current conditions were sufficiently bad, some children and young people felt positively about moving, to leave negative things behind. For instance, a move could take them close to friends [15] or they may have more space in the new property [15]. Quite often, however, negative impacts of moving seemed to offset any benefit [90].

Frequent moves could impact on children and young people’s health and wellbeing in other ways. Space might be even more squeezed by cardboard boxes in preparation for an impending move [15]. Some children reported having to leave beloved pets behind [90]. Time costs associated with moving meant less time for other activities [15]. Multiple moves, particularly across local authority boundaries, could impact the family’s access to services [41, 71], including health services [90], specialist healthcare required to manage children’s health conditions [83], and social services [85, 93].

One key impact that overlaid all of the above but was rarely mentioned was a lack of choice or control [109]. This was inherent in the families’ and children/young people’s accounts of their experiences of housing insecurity, through talk of not knowing where their next move would be or when, and having to move long distances away from the places they used frequently and the people who supported them. Even the journey into housing insecurity was often outside of families’ control, such as increases in rent, change in income, or eviction notices (see ‘Exposure’). Families often could not improve properties in poor condition because they could not afford repairs to properties in poor condition, so felt they had to live with these problems [90]. Some families avoided reporting problems to the landlord for fear of a rent increase or eviction (see ‘Exposure’). Children and young people in particular felt that they lacked control over their housing situation, and in some cases were not aware of reasons for moves [15].

### Outcomes

Several childhood health and wellbeing outcomes have been documented in relation to, and they are overwhelmingly negative. These consisted of mental health problems, physical health problems, tiredness, and stunted child development. Living in temporary housing, making multiple moves, and the instability and insecurity associated with anticipating a move, or being uncertain whether a move would be needed, had an obvious negative impact on the mental health of children and young people [41, 63, 79, 107], including in terms of self-harm [71, 96, 97, 107, 111], thoughts of suicide [71], anxiety [71, 90, 103, 111, 112, 115], and depression [110, 115]. Sometimes these problems manifested as physical pain [106], nightmares [84], night waking [107], or wetting the bed [63, 107, 111]. Stress, anger, isolation, fear, worry about the future (including about having to move again), worry about safety and acute distress were also reported [15, 63, 73, 79, 82, 84, 89, 90, 96, 109, 114, 115, 118]. One child with distress/mental health problems (as a result of having to make multiple moves) stopped eating properly (resulting in underweight and anaemia), and became socially withdrawn [79]. Another child reported weight loss and mental health problems due to worry about the future housing situation [95]. One study reported on stress and anxiety in children due to the trauma of losing their home and the emergency accommodation being unsuitable and temporary [111].

*‘My six year old has been going to the doctors because he’s developed a nervous tick since we’ve been in that room. He was constantly nervous all the time. He’s so unsettled still and he knows that we’re still not settled. He’s really anxious. He’s become violent […]’ (*[111]*, p.13)*

Sometimes children and young people’s mental health issues would be displayed through problematic behaviour such as withdrawal, stealing, smoking, drug-taking, aggressive behaviour, and running away [68, 71, 79, 84, 97, 107, 114, 115]. Teachers observed that younger children tended to get more withdrawn and older children and young people tended to get more angry and antagonistic, although the same child could cycle between these two states [115]. Separation anxiety was also reported [87, 111].

Children and young people also experienced physical health problems as a result of living in temporary accommodation, poor condition housing, and making multiple moves, including the development or exacerbation of asthma [69, 81, 90] and eczema [41, 81, 90, 111], stomach bugs [71], insect bites [112], infectious diseases [41, 109, 112], headaches [113], stomach aches [109, 113], exacerbation of long-term conditions [41, 75, 109], rashes and asthma as a result of damp [100], a dermatological condition as a result of living in a hotel [91], other physical symptoms in young children, such as coughing and vomiting [100] and musculoskeletal pain from sleeping in unsuitable places [102]. One study reported illness in a baby following a difficult birth, attributed to housing-related stress in the mother [83]. Rarer outcomes included weight gain due to a lack of cooking facilities and thus reliance on fast food, weight loss due to stress [79, 95] and head lice due to close contact with others [115]. Some properties presented risk of injury to babies and young children [41].

Tiredness was also reported, in relation to travelling a long distance to school and to visit family and friends [15, 66, 77, 102, 112, 115]. Tiredness also resulted from poor quality sleep due to the unsuitable nature of the accommodation (e.g., poor state of repair, overcrowded), sleeping on a sofa [102], and worrying about the housing situation [15, 41, 87, 109, 112, 114].

Impacts on the perceived development of young children were reported, in particular in relation to having no space to play, which impacted standing/walking and emotional development [87, 111], and multiple moves, which impacted on potty training and speech development [87, 111]. One study reported an impact on growth due refusal of solid food [113].

### Protective factors

Protective factors were not presented in the original conceptual framework. However, we identified specific protective factors that were perceived to lessen the impact of housing insecurity on wellbeing among children and adolescents. These included friendship, keeping the same school, normalising housing insecurity, home-making, having a plan, hope, protective parenting, and some interventions.

Friendship was a key protective factor. Retaining connections with friends and peer networks following moves was important [15, 90], and school facilitated this [114]. Indeed, another related strategy was to keep children and young people enrolled in the same school during and after moves, to retain some stability [15, 70, 90, 108].

Some sources noted that children and young people tended to normalise and destigmatise their housing insecurity as something to be expected given that the family is poor or receives benefits [15, 62, 90]. This response could be a coping/defence mechanism to try to deal with the negative impacts of being insecurely housed.

Another, more positive, coping strategy was to make the property feel more like a home. For instance, decorating the property could lead to children and young people feeling more settled and ‘at home’, even if the ultimate intention was to move [15]. Further coping strategies included having a plan of how things could go to keep anxiety at bay and retain some control [15], seeing the advantages of a location [15], and hoping for a better house next time, and/or hoping that the family would settle in a permanent home [15].

Parents also acted to protect children and young people from the negative impacts of housing insecurity, by concealing the full extent of their financial and housing problems [113], including children and young people in decision-making [70, 90] (for instance, allowing children and young people to influence their parents’ decisions on location, where there was a choice [70]), and presenting their situation as an adventure [114]. One study also documented parents taking their children out to parks to give them space to run around [91].

Lastly, some positive findings were reported by an evaluation of the Families Intervention Project (FIP), for families at risk of eviction due to anti-social behaviour [118]. Families that worked closely with a multi-agency team experienced increased housing security, reduced stress and anxiety, and fewer behavioural problems among the children [118]. Another study reported positive effects of a peer-led parenting programme on children’s behaviour, although it is unclear how this impacted on their health and wellbeing [64].

### Key findings relating to other populations

#### Families that have experienced domestic violence

Domestic violence could be a source of housing insecurity both for families who leave the family home to seek safety and for those who stay. Families that leave can end up moving multiple times (and frequently), perhaps initially to a refuge and then into other forms of temporary housing, with families experiencing uncertainty over when the next move would be [90, 105]. One study reported that experience of living in different places was perceived to be beneficial, although little detail was given, and this was offset by difficulty building peer networks [90]. In one family, the alternative to housing insecurity was for the children to be placed in local authority care, which was avoided through the children and other parent leaving the perpetrator [90].

Among families who stay in the family home (with the perpetrator leaving), housing insecurity could be created by the perpetrator refusing to pay the mortgage, leaving the family worried and uncertain:

‘*I’ve lost two stone, my son has lost ten pounds – he is only 15 – he is having counselling at school. It has just been a nightmare…He hasn’t paid the mortgage for a year because he wants to get me out so he can have the money…*’ ([95], p. 68).

Friendship was particularly impacted among this population. To prevent the perpetrator from finding them, children were not able to disclose personal information [63]. This made it difficult to form close friendships.

Parents reported a lack of support offered to children and young people, including services that they needed [80]. However, where support was offered to parents and children/young people who had moved to escape abuse in their previous home, this support could improve wellbeing [63, 79, 80], acting as a protective factor. Particular forms of useful support included a parenting course [79] and supportive staff and peers at hostels [80]. Hostels offered a feeling of safety due to closed-circuit television [80]. One study reported that refuge and hostel staff were perceived as helpful but powerless to keep families safe in some cases, although children and young people found it helpful to talk and open up to staff about their situation [63]. One intervention, the Sanctuary scheme, allowed people experiencing/at risk of domestic violence to remain in their own home, with additional security [95].

### Migrant, refugee and asylum seeker families

Migrant, refugee and asylum seeker families experienced similar forms of housing insecurity and similar impacts on everyday life and childhood health/wellbeing as did the general population. However, migrant/refugee families reported having to move suddenly, with very little notice (e.g., 48 h) [77, 82]. They also lacked support from services and assistance with housing from the local authority. Consequently, families would end up homeless and have to beg friends to let them sleep on their sofas [101].

Once homeless, families slept in unsuitable locations, such as on the night bus, in a church, and in the waiting room of the Accident and Emergency (A&E) department. This led to extreme tiredness; in some cases, children were too tired to attend school [102]. That type of homelessness was a particular feature of the experience of housing insecurity among this population.

*‘We had to keep going to McDonalds every night and we would also go to A&E. I would have to wear my school clothes and sleep like that.… They would say we have to sleep where the people wait but it’s just like lights […] The chairs were hard.’ (child aged 9) (*[102]*, p. 22)*

Other considerations specific to migrant/refugee/asylum seeker families were language barriers, which compounded the challenge of adjusting to a new area [82], and pressure to cook British food rather than food from their home country in communal facilities [106].

### Families forced to relocate due to demolition

Two papers identified from the database search examined experiences of relocation; families were living in local authority accommodation in Glasgow and experienced a forced move as the high-rise block of flats they lived in was due to be demolished [69, 70]. This forced location creates housing insecurity.

Despite the common source, however, housing insecurity was experienced in different ways by different families. One family reported not wanting to move as the children liked the area and their school and nursery, and one family was offered a flat but needed outdoor space [70]. Many families experienced the pre-relocation area as unsafe due to problematic behaviour in outdoor shared areas [69]. Because of this and no access to a private garden children lacked space to play [70]. Families also reported feeling shame in relation to the local area and the poor condition of their pre-relocation housing (a high-rise block of flats), and were keen to move to a less stigmatising area with better condition housing [69, 70].

Most families managed to relocate to areas close enough for their children and young people to attend the same schools. However two families changed schools [69, 70]. Children and young people felt shame and stigma relating to the local area and the flats themselves, with many young people reluctant to invite friends over, or others socialising in the corridor without inviting friends inside [70]. Thus, relocation could have positive impacts on families and children/young people. For three families, moving was a positive experience, with children and young people enjoying having a garden and growing to like their new neighbours and the area [69].

## Discussion

Although we anticipated potentially different experiences, impacts and outcomes relating to housing insecurity across the four populations, the evidence reviewed suggests many similarities. Some exposures were common to multiple populations, for instance, being evicted or having a forced move, living in temporary accommodation, experiencing overcrowding, exposure to problematic behaviour, poor condition/unsuitable property, and making multiple moves. Common impacts included social, school-related, psychological, financial and family wellbeing impacts, having to travel long distances to attend school and see friends, having to live in a property that was unsuitable or in a poor state of repair, overcrowded and often noisy, all of which could then further exacerbate housing insecurity. Outcomes reported across multiple populations included mental health problems (which could manifest in physical ways, for example, trouble eating and sleeping, or wetting the bed) and physical health problems such as skin complaints and asthma related to poor housing conditions. Protective factors common to multiple populations included friendship and support, staying at the same school, having hope for the future, and parenting practices. Pervasive throughout all populations and accounts was an overall lack of choice or control over the housing situation and poverty as a driving force.

These findings support and build upon previous literature that has examined the impact of housing insecurity on the health and wellbeing of children and young people, in terms of reduced mental and psychological wellbeing [21, 42, 43], ill health relating to homelessness or poor housing conditions [40, 41], and disrupted family processes [38]. Likewise, the findings build upon prior cohort studies that support a causal relationship with child health [38], by highlighting the details of the hardships faced by children and young people experiencing housing insecurity and exploring relationships between exposures, ‘less tangible’ impacts and health and wellbeing outcomes.

Many elements of the Children’s Society definition of housing insecurity were identifiable in our review findings. A key element of housing insecurity is financial insecurity [17, 19]; this was borne out in our findings where families were frequently exposed to high/rising costs of housing or reduced income. Indeed, our review found that families incurred additional costs due to multiple and/or frequent moves and/or moving into temporary accommodation. This could potentially increase financial insecurity, thus creating a vicious circle of housing insecurity and poverty. Having ‘a home that does not provide a sense of safety and security’ ([18], paragraph 3) was evident when children and young people reported not feeling safe in their accommodation, and relational insecurity was evident in families’ accounts of being moved far from friends, school and support networks.

In addition, we identified certain population-specific considerations. Families experiencing domestic violence faced a difficult choice between choosing to remain in the property and leaving the property, both with insecurity attached. Housing insecurity negatively impacted on friendships for all populations, however this could be potentially more challenging for those escaping domestic violence, due to the need to keep personal information confidential in order to maintain family safety.

Parents and children/young people in migrant, refugee and asylum seeker populations spoke of having very little notice before having to move out of a property, sometimes only 48 h. This created a housing emergency, captured in accounts of families becoming homeless and having to sleep in unsuitable places, such as the Accident and Emergency department waiting room or on a night bus. In some families, parents had no recourse to public funds, so even when children and young people were born in the UK, the family still ended up destitute and homeless, leading to significant worry.

A key factor in relocation was that families were forced to move by a particular date, as the high-rise block they lived in was scheduled for demolition. Many families desired a move, due to a lack of space, overcrowding, and unsafe outdoor spaces. However many did not want to leave behind social networks and schools in the community, and even some who wanted to move had difficulty finding a suitable property (e.g., for their family size).

A key challenge to synthesising the evidence was the complexity of the data, in particular the relationships between exposures and impacts. Factors that families initially experienced as exposures could then become impacts, and particular impacts could then worsen housing insecurity, in a cyclical fashion. For instance, overcrowded conditions could precipitate a move, but then the only property available may be in a poor state of repair, with intolerable living conditions, thus prompting a further move. Another key challenge in synthesising the qualitative evidence was that many elements of the experience of housing insecurity that were experienced simultaneously by children and young people have been artificially separated within the updated conceptual frameworks, making analysis problematic. For instance, those living in poor-condition temporary accommodation may want to move due the poor state of a property, but be worried about where they may end up next and whether children/young people will have to change schools and move far from friends. Such complexity has proved challenging to our overall synthesis. Policymakers and practitioners should be aware that the diagrams illustrating the hypothesised causal pathways simplify the multiple inter-related factors related to housing insecurity that impact on the wellbeing of children and young people. Identifiable common stresses including poverty, financial difficulties and debt, immigration/refugee status and domestic abuse will also exert direct significant effects on family wellbeing that prove difficult to separate from those directly due to housing insecurity.

### Limitations

#### Limitations of the evidence base

We have identified numerous literature sources, many rich with data relating to the experiences of children and young people, and synthesised these data into diagrams that illustrate hypothesised causal pathways within the original conceptual framework, with accompanying descriptions of the experiences of housing insecurity in children and young people. However, we cannot establish claims for the comprehensiveness of our diagrams that map hypothesised causal pathways from housing insecurity to childhood health/wellbeing based on the original conceptual framework. We mapped associations where they were present in the accounts of children/young people and other informants. However, the evidence base may have missed other potential associations, particularly for populations covered by a small number of studies.

Within the evidence base, accounts from parents or other informants proved extremely useful in examining the impacts of housing insecurity on the health and wellbeing of children and young people, particularly for younger children who are not able to yet articulate their experiences and feelings. Nevertheless, such accounts proved an insufficient substitute for rich and nuanced data directly from the children and young people themselves. Our public involvement group have informed us that children and young people may find it difficult to talk about their housing situation, and noted that we did not identify any research that explicitly examined the perspectives of young people who provide care for a parent.

Likewise, little available information relates to the health and wellbeing of children and young people, and it is difficult to establish whether the evidence we have reviewed has captured all relevant health and wellbeing experiences. The majority of the accounts of young people focused on the impacts (or intermediate outcomes) of housing insecurity, which means that we have been able to present a rich picture of these ‘less tangible’ impacts, but also that the links from these impacts to health and wellbeing outcomes is less well understood. For instance, our public involvement group noted that we had not reported any evidence relating to bullying as a result of experiencing housing insecurity.

### Strengths and limitations of the review

Strengths of our review method include the prior use of a conceptual model, developed in consultation with stakeholders and topic experts, and examination of key policy documents, which guided the process of synthesis. Synthesis was thus both deductive (i.e., informed by the a priori conceptual model) and inductive (i.e., conducted using established thematic synthesis methods), which allowed for an organised and yet rich and nuanced picture of the impacts of housing insecurity on health and wellbeing among children and young people in the UK. The review was conducted by an established team of experienced reviewers and a methodologist.

A key limitation is that literature sources were far more plentiful than anticipated, including numerous long and detailed reports identified through grey literature searching. While this enhanced the richness of the dataset, it also expanded the review workload, leading to additional time constraints. Limited time and resource could be allocated for double-checking full texts (in particular in the grey literature) and extractions, and thus only a sample were double-checked. Time constraints also prevented citation searches of key included studies. Nevertheless, such an approach remains consistent with established rapid review methods with minimal consequences for missing or mis-reported evidence [50–52]. Time and resource constraints also prohibited examination of how experiences may differ according to location within the UK.

### Implications for policy

It is important that decisions made about housing at a national and local level reflect the impacts that insecure housing can have on children and young people, and ensures that housing insecurity is prevented in the first place. The current review findings suggest that policies should focus on reducing housing insecurity in its totality among families. One way to do this is to focus on eviction, which is a significant cause of instability and a leading cause of households seeking homelessness assistance [25]. This could include ending no-fault evictions, as has been done in Scotland for private renters since 2017 and as proposed, but yet to be introduced by the UK government in 2019. Scotland’s introduction of longer tenancy agreements with the removal of no-fault evictions may also facilitate families being able to settle and reduce the need for multiple moves. Similarly, legislating for minimum standards in the private rented sector, as currently being explored [119], will protect children and young people from being exposed to unhealthy and dangerous conditions.

Other changes could include (1) stipulating minimum requirements for space in family properties and minimum requirements for property conditions; (2) advocating for families living in the private rental sector to improve their housing situation; (3) reducing the use of short-term tenancies so families are not required to make multiple moves; (4) providing affordable housing options that give families more choice; and (5) engaging families in the design of systems and services that meet their housing needs. Addressing poverty more widely should also help to alleviate housing insecurity among families in the UK, as much of the evidence reported on how poverty initiated and/or exacerbated housing insecurity, for instance, by restricting choice and by increasing worry. However, any changes will need adequate support for enforcement, something made clear by the limited effectiveness of policy introduced to protect people from revenge/retaliatory eviction [97, 120–122], improve the quality and suitability of temporary accommodation, and, where possible, reduce the need for temporary accommodation through preventative measures.

Among families escaping domestic violence, support systems are needed to avoid destitution caused by the perpetrator (e.g., not paying the mortgage). There should also be systems in place to ensure that families are housed in a permanent residence as soon as possible following the initial placement in emergency temporary accommodation after leaving the family home, with as few moves as possible. Appropriate support with housing should be made available to refugee/asylum seeker/migrant families, including those where the parents have no recourse to public funds, and short-notice and long-distance moves should be avoided, particularly where these take families away from their support systems and communities.

### Implications for practice

Where possible, interventions to reduce or eliminate housing insecurity should be implemented. Where this is not possible, interventions should focus on reducing the impact of housing insecurity, for instance, by ensuring long journeys can be avoided, that accommodation is of a decent standard, and by providing adequate support to families and children young people. Practitioners who work to house families should prioritise stable, suitable and good quality housing. Practitioners who interact with children and young people experiencing housing insecurity and homelessness (e.g., clinicians, teachers, social workers) should recognise the complexity of the children and young people’s experiences, including how the situation and circumstances (e.g., escaping domestic violence, migration status) might impact on their health and wellbeing, and that impacts vary on an individual basis, particularly in assessments and family support plans. A multiagency approach should be utilised with families to mitigate the impacts of housing insecurity, poor housing conditions or unsuitable housing. Practitioners should consider the impacts of continuity of school, support and services, and the need for mental health support, parenting and counselling, for instance through providing support with transport to enable children and young people to stay at their current school, and support to maintain friendships. All those working with children/young people and families experiencing housing insecurity should consider ways to offer them optimal choice and control over situations that affect them.

All practitioners and professionals (e.g., teachers) who work with children and young people from families who have escaped domestic violence should ensure that the children and young people are receiving appropriate support from all relevant services, and that appropriate safety measures are in place to protect the family from the perpetrator.

### Research recommendations

Future qualitative research could focus explicitly on the health and wellbeing of children and young people experiencing housing insecurity, and how they link with the impacts and outcomes identified in the current review. In particular, research could explore how the health and wellbeing of children and young people are affected by the impacts of housing insecurity on friendships, education, food and hygiene, financial impacts, long journeys, overcrowding, perceived safety, and access to services. Further qualitative research could examine the impact of interventions to address housing insecurity among families in the UK. Interventions with a participatory component that seek to ensure that the voices of children and young people remain central should be prioritised for further research. The voices of specific groups of young people who are likely to be marginalised (e.g., young carers) could be explored in future research. Future qualitative research should report methods of recruitment and data collection and analysis clearly and transparently, and should incorporate meaningful research reflexivity.

## Conclusions

Housing insecurity has a profound impact on children and young people in families in the UK. Such housing insecurity can take many forms and result from often inter-related situations that are fundamentally connected to poverty. The resultant housing insecurity can have multiple (often simultaneous) impacts, including those that relate to educational, psychological, financial and family wellbeing impacts, having to travel long distances to attend school and see friends, and having to live in unsuitable, poorly repaired, overcrowded or noisy properties, any of which further exacerbate housing insecurity. Negative experiences can impact on health and wellbeing, in terms of mental health problems (which could manifest in physical ways) and physical health problems, as well as tiredness and developmental issues. Some experiences and situations can lessen the impact of housing insecurity on the health and wellbeing of children and young people. Negative impacts of housing insecurity on health and wellbeing may be further compounded by specific situations and life circumstances, such as escaping domestic violence, being a migrant, refugee or asylum seeker (or having a parent with that status), or experiencing a forced relocation due to housing demolition.

## Supplementary Information

Supplementary Material 1.

Supplementary Material 2.

Supplementary Material 3.

Supplementary Material 4.

## Data Availability

All data presented in this review were already published, either in an academic journal, or a report that is publicly available. Search strings are available in Additional File 1. Data extracted from the published papers and reports included in the current study are available from the corresponding author on request.

## Abbreviations

A&E: Accident and Emergency (Department)
AACODS: Authority, Accuracy, Coverage, Objectivity, Date, Significance
ASSIA: Applied Social Sciences Index and Abstracts
B&B: Bed and Breakfast (accommodation)
CASP: Critical Appraisal Skills Programme
COVID-19: Coronavirus Disease 2019
FIP: Families Intervention Project
UK: United Kingdom
IBSS: International Bibliography of the Social Sciences
NIHR: National Institute for Health and Care Research
PROSPERO: International prospective register of systematic reviews

## Footnotes

1 the main social security payment in the UK; for more information see https://www.gov.uk/universal-credit
