# Supplementary material for: Exploring the impact of housing insecurity on the health and wellbeing of children and young people in the United Kingdom: a qualitative systematic review
Source: BMC Public Health. 2024 Sep 9;24:2453. doi: 10.1186/s12889-024-19735-9 (PMC11385840; doi:10.1186/s12889-024-19735-9)
Supplement: Supplementary file 1 — Supplementary Material 1. [file 12889_2024_19735_MOESM1_ESM.docx]

## **Exploring the impact of housing insecurity on the health and wellbeing of children and young people in the United Kingdom: a qualitative systematic review**

## **Additional File 1: Simplified database search strategy**

File name: Additional File 1 - Search strategy

File type: Microsoft Word Document (.docx)

Title of data: Simplified database search strategy

Description of data: search strings used in the database searches, for MEDLINE, EMBASE and PsycInfo.

**MEDLINE via Ovid (searched 8^th^ April 2022)**

Ovid MEDLINE(R) and Epub Ahead of Print, In-Process, In-Data-Review & Other Non-Indexed Citations, Daily and Versions

1. ((hous* or flat or flats or apartment* or accommodation or home* or tenan* or tenure or residen* or abode or lodg*) adj3 (fixed or secur* or insecur* or unstab* or instab* or stable or stabil* or temporar* or transitional or transient or precar* or mobil* or unsuitab* or unfit* or inadequa*)).mp. 
2. ((rent* or mortgag*) adj3 (worr* or concern* or afford* or unafford* or pay* or debt* or arrear*)).mp. 
3. (homeless* or sofa surf* or (bed adj2 breakfast*)).mp. 
4. (evict* or (forc* adj2 (mov* or relocat*))).mp. 
5. 1 or 2 or 3 or 4 
6. (poverty or disadvantage or underserved or low* income or deprivation or austerity or unemploy* or universal credit or benefit claim* or free school meal*).mp. 
7. (hous* or home* or accommodation or residen* or abode* or flat or flats or apartment* or lodg*).mp. 
8. 6 and 7 
9. (child* or infant* or adolescen* or teenage* or school* or family or families or parent*).mp. 
10. (interview: or experience:).mp. or [qualitative.tw](http://qualitative.tw/). 
11. (5 or 8) and 9 and 10 
12. limit 11 to yr="2000 -Current"

13. exp Great Britain/ 
14. (national health service* or nhs*).ti,ab,in. 
15. (english not ((published or publication* or translat* or written or language* or speak* or literature or citation*) adj5 english)).ti,ab. 
16. (gb or "g.b." or britain* or (british* not "british columbia") or uk or "u.k." or united kingdom* or (england* not "new england") or northern ireland* or northern irish* or scotland* or scottish* or ((wales or "south wales") not "new south wales") or welsh*).ti,ab,jw,in. 
17. 13 or 14 or 15 or 16 
18. (exp africa/ or exp americas/ or exp antarctic regions/ or exp arctic regions/ or exp asia/ or expoceania/) not (exp great britain/ or europe/) 
19. 17 not 18 
20. 11 and 19

**EMBASE via Ovid (searched 8^th^ April 2022)**

| 1. | ((hous* or flat or flats or apartment* or accommodation or home* or tenan* or tenure or residen* or abode or lodg*)  adj3 (fixed or secur* or insecur* or unstab* or instab* or stable or stabil* or temporar* or transitional or transient  or precar* or mobil* or unsuitab* or unfit* or inadequa*)).mp. |
| --- | --- |
| 2. | ((rent* or mortgag*) adj3 (worr* or concern* or afford* or unafford* or pay* or debt* or arrear*)).mp. |
| 3. | (homeless* or sofa surf* or (bed adj2 breakfast*)).mp. |
| 4. | (evict* or (forc* adj2 (mov* or relocat*))).mp. |
| 5. | 1 or 2 or 3 or 4 |
| 6. | (poverty or disadvantage or underserved or low* income or deprivation or austerity  or unemploy* or universal credit or benefit claim* or free school meal*).mp. |
| 7. | (hous* or home* or accommodation or residen* or abode* or flat or flats or apartment* or lodg*).mp. |
| 8. | 6 and 7 |
| 9. | (child* or infant* or adolescen* or teenage* or school* or family or families or parent*).mp. |
| 10. | experience:.mp. or interview:.tw. or qualitative:.tw. |
| 11. | 5 and 8 and 9 and 10 |
| 12. | limit 11 to english language |
| 13. | limit 12 to yr="2000 -Current" |
| 14. | (UK or united kingdom or britain or england or scotland or wales or ireland).lo. |
| 15. | 13 and 14 |
| 16. | 13 not 14 |
| 17. | limit 13 to dissertation |
| 18. | 13 not 17 |

**PsycINFO via Ovid (searched 8^th^ April 2022)**

| 1. | ((hous* or flat or flats or apartment* or accommodation or home* or tenan* or tenure or residen* or abode or lodg*)  adj3 (fixed or secur* or insecur* or unstab* or instab* or stable or stabil* or temporar* or transitional or transient  or precar* or mobil* or unsuitab* or unfit* or inadequa*)).mp. |
| --- | --- |
| 2. | ((rent* or mortgag*) adj3 (worr* or concern* or afford* or unafford* or pay* or debt* or arrear*)).mp. |
| 3. | (homeless* or sofa surf* or (bed adj2 breakfast*)).mp. |
| 4. | (evict* or (forc* adj2 (mov* or relocat*))).mp. |
| 5. | 1 or 2 or 3 or 4 |
| 6. | (poverty or disadvantage or underserved or low* income or deprivation or austerity or unemploy* or universal credit  or benefit claim* or free school meal*).mp. |
| 7. | (hous* or home* or accommodation or residen* or abode* or flat or flats or apartment* or lodg*).mp. |
| 8. | 6 and 7 |
| 9. | (child* or infant* or adolescen* or teenage* or school* or family or families or parent*).mp. |
| 10. | experience:.mp. or interview:.tw. or qualitative:.tw. |
| 11. | 5 and 8 and 9 and 10 |
| 12. | limit 11 to english language |
| 13. | limit 12 to yr="2000 -Current" |
| 14. | (UK or united kingdom or britain or england or scotland or wales or ireland).lo. |
| 15. | 13 and 14 |
| 16. | 13 not 14 |
| 17. | limit 13 to dissertation |
| 18. | 13 not 17 |
