# Supplementary material for: Exploring the impact of housing insecurity on the health and wellbeing of children and young people in the United Kingdom: a qualitative systematic review
Source: BMC Public Health. 2024 Sep 9;24:2453. doi: 10.1186/s12889-024-19735-9 (PMC11385840; doi:10.1186/s12889-024-19735-9)
Supplement: Supplementary file 2 — Supplementary Material 2. [file 12889_2024_19735_MOESM2_ESM.docx]

## **Exploring the impact of housing insecurity on the health and wellbeing of children and young people in the United Kingdom: a qualitative systematic review**

**Additional File 2: Grey literature searches**

File name: Additional File 2 - Grey literature searches

File type: Microsoft Word Document (.docx)

Title of data: Supplementary Table 1: Sources searched and relevant documents identified in grey literature searches

Description of data: Details of organisations and their websites that were searched, the pathways followed within the website searches, approach to screening in each case, number of documents scanned and relevant records.

**Supplementary Table 1: Sources searched and relevant documents identified in grey literature searches**

| **Organisation** | **Website** | **Pathway followed** | **Approach to screening** | **Number of documents scanned** | **Number of relevant records** |
| --- | --- | --- | --- | --- | --- |
| Children’s Commissioner for England | <https://www.childrenscommissioner.gov.uk/> | Reports  Searched category=Any, Topic=Any, Newest First | Reports - newest first. Scanned titles and opened report of anything relevant and searched “hous” and “mov” using the ‘Find’ function. | 46 | 6 |
| The Children’s Society | <https://www.childrenssociety.org.uk/> | What We Do 🡪 Blogs 🡪 The Good Childhood Report / Our campaigns for children / Our impact / Young people's stories  Information for 🡪 Professionals 🡪 Resources and publications | Within What We Do 🡪 Blogs 🡪 The Good Childhood Report: screened the one report available.  Within Information for 🡪 Professionals 🡪 Resources and publications: searched for ‘all resources’ on ‘child poverty’ for ‘all years’. | 8 | 3 |
| AYPH / Young People’s Health Partnership | <https://www.youngpeopleshealth.org.uk/yphp> | Resources 🡪 Health Inequalities & Social Determinants  AYHP generally  Homepage | Screened the resources. | 2 | 0 |
| The Health Foundation | <https://www.health.org.uk/> | Home > publications > view all publications > topics: children and young people | Word search = ‘hous’ & ‘home’ | 2 | 0 |
| National Children’s Bureau | <https://www.ncb.org.uk/> | Resources 🡪 Major reports  Resources 🡪 All resources [searched for ‘hous’] | Screened titles of first 12 pages on website then did a search for ‘hous’. | 10 | 0 |
| Child Poverty Action Group | <https://cpag.org.uk/> | Policies and campaigns 🡪 briefings and reports [Filter by topic: housing] | Screened titles and then screened full texts of potentially relevant ones. | 21 | 4 |
| Royal College of Paediatrics and Child Health | <https://www.rcpch.ac.uk/> | Initially tried Resources but the list was difficult to screen.  Therefore, tried Topics 🡪 child poverty | Screened full texts in poverty section. | 5 | 2 |
| Children and Young People’s Mental Health Coalition | <https://cypmhc.org.uk/> | Publications [hand-searched list] | Hand-searched list of reports by title, then searched full text of any potentially relevant ones for “hous”, “home” and “mov” using Find function. | 8 | 0 |
| Homeless link | <https://www.homeless.org.uk/> | Our Work 🡪 Our research 🡪 Our current research 🡪 Young and homeless research / Supported housing and hostels / Welfare reform research / Homeless Health Needs / Audit / Move-on / Prevention | Screened titles, then searched full text of any potentially relevant ones for “family”, using Find function, or skimmed document. | 5 | 0 |
| The Queen’s Nursing Institute | <https://www.qni.org.uk/> | Homeless and Inclusion Health Programme 🡪 Link to published paper [‘Health visiting with homeless families during the COVID-19 pandemic’] / Facts about homelessness and health / Learn from different case studies / Innovation projects / Read guides and reports | Screened titles of relevant reports or links, then skimmed full texts of those. | 16 | 0 |
| Health and Wellbeing Alliance | <https://www.ncvo.org.uk/vcse-health-and-wellbeing-alliance> | Policy and Research 🡪 Research Conference [Page not found] / team of researchers | Could not find any reports of research findings | 0 | 0 |
| Project 17 | <https://www.project17.org.uk/> | Resources  Policy 🡪 Briefings and reports 🡪 Briefings / Reports  News | Screened titles, and screened documents that looked relevant. | 4 | 2 |
| Maternity Action Migrant Women’s Rights Service | <https://maternityaction.org.uk/migrant-womens-rights-service/> | What we do → Reports and publications | Screened titles, and screened documents that looked relevant. Searched for “hous” and “home” in longer reports using Find function. | 12 | 1 |
| Race Equality Foundation | <https://raceequalityfoundation.org.uk/> | Our work 🡪 Housing 🡪 Better Housing Project 🡪 Briefings and Reports / Blog / Briefings  [Searched ‘housing’ and ‘homelessness’ from the drop-down list of categories] | Blog - clicked on blog posts related to housing and screened.  Briefings - ‘housing’ - screened titles, screened relevant reports and references. | 26 | 1 |
| Runnymede Trust | <https://www.runnymedetrust.org/> | See Our Latest Publications  Our work 🡪 Publications [Filter by category = ‘Housing’] | Screened titles, screened text of relevant reports (shorter), searched longer reports for “hous”, “home” and “mov” using Find function. | 3 | 0 |
| Trust for London | <https://www.trustforlondon.org.uk/> | Publications | Screened titles, screened relevant reports | 16 | 0 |
| Shelter | <https://england.shelter.org.uk/> | Policy & Research  [Checked Publications (just housing costs handbooks); checked policy library (briefings); Sustain research project (final report)]  **briefings seemed to draw on reports - so just looked at reports.  **revised search strategy due to volume of literature. Added ‘insecure’ to the search bar = 37 results. | Screened titles (initially of 200 documents relating to family and children and then the narrower search within these of 37 relating to insecurity), then screened full text of potentially relevant reports. | 23 | 12 |
| British Medical Association | <https://www.bma.org.uk/> | Searched website for the term ‘housing’ | 34 hits from searching website with term “housing”, screened titles of these | 3 | 0 |
| Toynbee Hall | <https://www.toynbeehall.org.uk/> | Research 🡪 Research and evaluation reports | Screened titles listed under ‘Research’.  Screened titles listed under ‘Browse our research and evaluation reports’. | 4 | 0 |
| Renters’ Reform Coalition | <https://www.rentersreformcoalition.co.uk/> | Policy [heading] | Only one report was available to view – screened that report. | 1 | 1 |
| Joseph Rowntree Foundation | <https://www.jrf.org.uk/> | Search ‘housing’  Filters: (301 documents)  1. Reports  2. Housing  [to start of 2007] | Searched back to 2007. Screened full texts. | 301 | 3 |
| Centrepoint | <https://centrepoint.org.uk/> | What we do 🡪 Policy and research 🡪 Research reports / Briefings  Blog | Screened titles listed under ‘Research reports’, ‘briefings and ‘blog’’. Screened document or searched for ‘child’, ‘family’, ‘baby’, ‘toddler’, ‘son’ and ‘daughter’. | 6 | 0 |
| The Young Women’s Trust | <https://www.youngwomenstrust.org/> | Research Centre 🡪 Explore our research reports | No relevant search options from drop-down menu for research reports, so screened titles. Skimmed document or searched for ‘hous’, ‘home’ and ‘mov’. | 7 | 1 |
| What Works Wellbeing | <https://whatworkswellbeing.org/> | Searched website for ‘housing’ | Website search for ‘housing’ produced three results. Screened each report.  One report cited another report (Homes, Health and Covid-19, by the Centre for Ageing Better https://ageing-better.org.uk/publications/homes-health-and-covid-19), which contained references to eight potentially relevant sources, which were all screened.  .  Screened another report by searching for ‘child’, ‘family’ and kid’, using the Find function. Followed up 3 references from that report - none relevant. | 33 | 1 |
| Cache | <https://housingevidence.ac.uk/> | Publications  Type = reports  Keyword = insecurity | Screened full texts. | 15 | 0 |
| Groundswell | <https://groundswell.org.uk/> | Research 🡪 Current projects 🡪 Benefits for health  Research 🡪 Current projects 🡪 Fulfilling Lives Lambeth, Southwark and Lewisham  Research 🡪 Current projects 🡪 ListenUp!  Research 🡪 Past projects 🡪 The Escape Plan  Research 🡪 Past projects 🡪 Out of Pain - Breaking the cycle of physical pain and homelessness  Research 🡪 Past projects 🡪 Room to Breathe  Research 🡪 Past projects 🡪 Universal Credit – the health impacts for people who are experiencing homelessness  Research 🡪 Past projects 🡪 Women, homelessness and health  Research 🡪 Publications | Screened titles of research projects and publications, screened reports of ones with titles that could be relevant. Searched some reports for the terms ‘child’, ‘family’, ‘kid’, ‘son’, ‘daughter’ using the Find function. | 22 | 0 |
| Chartered Institute of Housing | <https://www.cih.org/> | News → Publications  Title sifted first 5 pages, then narrowed down to the categories ‘affordability and benefits’, ‘health’ and ‘homelessness’ | Screened titles of publications, screened reports of ones with titles that could be relevant. | 44 | 1 (referenced from a report on the website) |
| Peabody | <https://www.peabody.org.uk/> | Searched website for ‘report’ [166 hits]  What we do 🡪 Research 🡪 Our research publications | Screened titles of reports (the 166 records found searching the word ‘report’), skimmed reports of ones with titles that suggested potential relevance. | 17 | 0 |
| Centre for Housing Policy (York Uni) | <https://www.york.ac.uk/chp/> | Publications | Screened titles of publications, screened full texts of ones with titles suggested potential relevance. | 15 | 0 |
| New Economics Foundation | <https://neweconomics.org/> | Publications | Screened titles of publications, screened full texts of ones with titles suggested potential relevance. | 12 | 0 |
| Crisis | <https://www.crisis.org.uk/> | Knowledge hub | Knowledge hub - screened titles of documents, screened full texts of ones with titles that could be relevant. For long reports, searched for ‘child’ and ‘family’ using Find function. | 39 | 2 |
