# Supplementary material for: Exploring the impact of housing insecurity on the health and wellbeing of children and young people in the United Kingdom: a qualitative systematic review
Source: BMC Public Health. 2024 Sep 9;24:2453. doi: 10.1186/s12889-024-19735-9 (PMC11385840; doi:10.1186/s12889-024-19735-9)
Supplement: Supplementary file 3 — Supplementary Material 3. [file 12889_2024_19735_MOESM3_ESM.docx]

## **Exploring the impact of housing insecurity on the health and wellbeing of children and young people in the United Kingdom: a qualitative systematic review**

## **Additional File 3: Full text excluded studies**

File name: Additional File 3 - Studies excluded at full text

File type: Microsoft Word Document (.docx)

Title of data: Supplementary Table 2: Studies excluded at full text from the database and reference list searches

Description of data: Bibliographic references and reasons for exclusion for all sources excluded at full text screening, from sources identified through the database and reference list searches.

**Supplementary Table 2: Studies excluded at full text from the database and reference list searches**

| **Paper** | **Reason** |
| --- | --- |
| The cuts hit home: austerity in Oxford. OpenDemocracy. London: OpenDemocracy, 2015. | No data |
| The human beings that UK government 'forgot'. OpenDemocracy. London: OpenDemocracy, 2016. | Not related to housing insecurity |
| About Shine A Light. OpenDemocracy. London: OpenDemocracy, 2019. | No data |
| Why the NHS Plan needs to be far more ambitious to tackle inequality. OpenDemocracy. London: OpenDemocracy, 2019. | Not qualitative |
| Ablewhite J, Kendrick D, Watson M, et al. Maternal perceptions of supervision in pre-school-aged children: a qualitative approach to understanding differences between families living in affluent and disadvantaged areas. Primary Health Care Research and Development 2015;16(4):346-55. doi: 10.1017/s1463423614000218 | Nothing on HI |
| Ablewhite J, Kendrick D, Watson M, et al. The other side of the story - maternal perceptions of safety advice and information: a qualitative approach. Child Care Health and Development 2015;41(6):1106-13. doi: 10.1111/cch.12224 | Nothing on HI |
| Abrams EM, Greenhawt M, Shaker M, et al. The COVID-19 pandemic: Adverse effects on the social determinants of health in children and families. Annals of allergy, asthma & immunology : official publication of the American College of Allergy, Asthma, & Immunology 2022;128(1):19-25. doi: https://dx.doi.org/10.1016/j.anai.2021.10.022 | Not UK (literature review / summary focusing on US) |
| Aceves-Martins M, Cruickshank M, Fraser C, et al. Child food insecurity in the UK: a rapid review. 2018 doi: https://dx.doi.org/10.3310/phr06130 | Not related to housing insecurity |
| Adams EA, Parker J, Jablonski T, et al. "It's been up and down"-perspectives on living through COVID-19 for individuals who experience homelessness: a qualitative study. The Lancet 2021;398(Supplement 2):S6. doi: https://dx.doi.org/10.1016/S0140-6736%2821%2902549-6 | Not related to housing insecurity (conference abstract, themes don’t relate to housing or income precarity) |
| Adams J. Tackling regional disparity: PPR. *New Economy* 2004;11(1):45-49. | Not related to housing insecurity |
| Ali N, Whitham B. Racial Capitalism, Islamophobia, and Austerity. *International Political Sociology* 2021;15(2):190-211. doi: 10.1093/ips/olaa023 | Not related to housing insecurity |
| Alkahtani S, Cherrill J, Tambe P, et al. Children's access to medicines. Archives of Disease in Childhood 2012;97(5):e19. doi: http://dx.doi.org/10.1136/archdischild-2012-301728.39 | Conference abstract, nothing on HI |
| Andersen K. Universal Credit, gender and unpaid childcare: Mothers' accounts of the new welfare conditionality regime. *Critical Social Policy* 2020;40(3):430-49. doi: 10.1177/0261018319856487 | Not related to housing insecurity |
| Anderson MR, Salisbury AL, Uebelacker LA, et al. Stress, coping and silver linings: How depressed perinatal women experienced the COVID-19 pandemic. Journal of Affective Disorders 2022;298:329-36. doi: https://dx.doi.org/10.1016/j.jad.2021.10.116 | Not related to housing insecurity |
| Anonymous. Bricks and mortar or flesh and blood? Community Care. Sutton: Mark Allen Group Ltd, 2007:16-18. | Not research (commentary – discussion between two social commentators) |
| Anonymous. FAMILIAR FAILINGS? Community Care. Sutton: Mark Allen Group Ltd, 2007:30-31. | Not research (commentary on a film about HI) |
| Anonymous. Impact of housing conditions on health and well-being. Community Care. Sutton: Mark Allen Group Ltd, 2007:24-25. | Not research (commentary / non-systematic review, no relevant evidence) |
| Anonymous. LOOK WHO'S TALKING. Community Care. Sutton: Mark Allen Group Ltd, 2005:54-55. | Not research (commentary – interview with mayor of London) |
| Anonymous. THE REAL ISSUE OF POVERTY. Community Care. Sutton: Mark Allen Group Ltd, 2005:5. | Not research (commentary) |
| Anonymous[. Public Health Science 2019](https://www-sciencedirect-com.sheffield.idm.oclc.org/journal/the-lancet/vol/394/suppl/S2). *The Lancet* 2019;394(Supplement 2):S1-S104. | Conference abstract list – nothing relevant |
| Anonymous. SLIPPING THROUGH THE NET. *Midwives* 2020;23:16-20. | Not available |
| Astrup J. CR£DIT CRUNCH: THE JOURNAL OF THE HEALTH VISITORS' ASSOCIATION. *Community Practitioner* 2019;92(2):14-17. | Not research (commentary) |
| Auchincloss M. KEYS TO THE DOOR. Planning. London: Haymarket Business Publications Ltd., 2008:16-17. | Not research (commentary) |
| Backett-Milburn KC, Wills WJ, Gregory S, et al. Making sense of eating, weight and risk in the early teenage years: Views and concerns of parents in poorer socio-economic circumstances. *Social Science & Medicine* 2006;63(3):624-35. doi: 10.1016/j.socscimed.2006.02.011 | Not related to housing insecurity |
| Barnes MC, Gunnell D, Davies R, et al. Understanding vulnerability to self-harm in times of economic hardship and austerity: a qualitative study. *Bmj Open* 2016;6(2) doi: 10.1136/bmjopen-2015-010131 | Not related to children |
| Bispham J. Wake up to the housing crisis blighting education. *The Times Educational Supplement* 2016(5182) | No details, no data |
| Bond H. December IJPP: Needle exchanges, and homeless women and children. Pharmaceutical Journal 2004;273(7328):828. | Not available |
| Bone J, O'Reilly K. No place called home: the causes and social consequences of the UK housing 'bubble'. *British Journal of Sociology* 2010;61(2):231-55. doi: 10.1111/j.1468-4446.2010.01311.x | No data on impact of HI on children’s health/wellbeing |
| Bowstead JC. Forced migration in the United Kingdom: women's journeys to escape domestic violence. *Transactions of the Institute of British Geographers* 2015;40(3):307-20. doi: 10.1111/tran.12085 | No data on impact of HI on children’s health/wellbeing |
| Bowstead JC. Spaces of safety and more-than-safety in women's refuges in England. *Gender Place and Culture* 2019;26(1):75-90. doi: 10.1080/0966369x.2018.1541871 | No focus on HI |
| Bradley C, McGowan J, Michelson D. How Does Homelessness Affect Parenting Behaviour? A Systematic Critical Review and Thematic Synthesis of Qualitative Research. *Clinical Child and Family Psychology Review* 2018;21(1):94-108. doi: 10.1007/s10567-017-0244-3 | Review – checked, all studies conducted in USA |
| Bradley T, Cupples ME, Irvine H. A case control study of a deprivation triangle: teenage motherhood, poor educational achievement and unemployment. *International journal of adolescent medicine and health* 2002;14(2):117-23. | Not qualitative |
| Brenisin K, Akinwande E, Trumm A, et al. The impact of inequality on mental illness: thematic analysis on clinical notes. Journal of Forensic Practice 2021;23(4):360-71. doi: http://dx.doi.org/10.1108/JFP-06-2021-0037 | Not available – only abstract |
| Briggs L, Lake AA. Exploring school and home food environments: perceptions of 8-10-year-olds and their parents in Newcastle upon Tyne, UK. *Public Health Nutrition* 2011;14(12):2227-35. doi: 10.1017/s1368980011001984 | Not related to housing insecurity |
| Brown CS, Lloyd S, Murray SA. Using consecutive Rapid Participatory Appraisal studies to assess, facilitate and evaluate health and social change in community settings. *Bmc Public Health* 2006;6 doi: 10.1186/1471-2458-6-68 | Not related to housing insecurity |
| Brown ED, Low CM. Chaotic Living Conditions and Sleep Problems Associated With Children's Responses to Academic Challenge. Journal of Family Psychology 2008;22(6):920-23. doi: http://dx.doi.org/10.1037/a0013652 | Not qualitative |
| Burgess DA, Phifer LW. Students exposed to domestic violence. Supporting and educating traumatized students: A guide for school-based professionals 2013:129-38. | Book – also population not relevant |
| Burstrom B, Whitehead M, Clayton S, et al. Health inequalities between lone and couple mothers and policy under different welfare regimes - the example of Italy, Sweden and Britain. Social science & medicine (1982) 2010;70(6):912-20. doi: https://dx.doi.org/10.1016/j.socscimed.2009.11.014 | Not qualitative |
| Butler T. Living in the bubble: Gentrification and its 'others' in north London. *Urban Studies* 2003;40(12):2469-86. doi: 10.1080/0042098032000136165 | Not related to housing insecurity |
| Buu A, DiPiazza C, Wang J, et al. Parent, family, and neighborhood effects on the development of child substance use and other psychopathology from preschool to the start of adulthood. Journal of Studies on Alcohol and Drugs 2009;70(4):489-98. doi: http://dx.doi.org/10.15288/jsad.2009.70.489 | Not UK  Not qualitative |
| Canvin K, Jones C, Marttila A, et al. Can I risk using public services? Perceived consequences of seeking help and health care among households living in poverty: qualitative study. Journal of epidemiology and community health 2007;61(11):984-9. | Not related to housing insecurity |
| Canvin K, Marttila A, Burstrom B, et al. Tales of the unexpected? Hidden resilience in poor households in Britain. Social science & medicine (1982) 2009;69(2):238-45. doi: https://dx.doi.org/10.1016/j.socscimed.2009.05.009 | Not related to housing insecurity |
| Chan C. The quality of life of women of Chinese origin. Health & Social Care in the Community 2000;8(3):212-22. doi: 10.1046/j.1365-2524.2000.00243.x | Not related to housing insecurity |
| Chanchlani N, Buchanan F, Gill PJ. Addressing the indirect effects of COVID-19 on the health of children and young people. CMAJ 2020;192(32):E921-E27. doi: http://dx.doi.org/10.1503/cmaj.201008 | Not related to housing insecurity |
| Cheetham M, Moffatt S, Addison M, et al. Impact of Universal Credit in North East England: a qualitative study of claimants and support staff. Bmj Open 2019;9(7) doi: 10.1136/bmjopen-2019-029611 | No data on impact of HI on children’s health/wellbeing |
| Christie N, Ward H, Kimberlee R, et al. Understanding high traffic injury risks for children in low socioeconomic areas: a qualitative study of parents' views. Injury Prevention 2007;13(6):394-97. doi: 10.1136/ip.2007.016659 | Nothing on HI |
| Clark AF, Barrett L, Kolvin I. Inner city disadvantage and family functioning. European Child & Adolescent Psychiatry 2000;9(2):77-83. doi: 10.1007/s007870050001 | Not qualitative |
| Clarke-Jones J. Moving forces. Nursing standard (Royal College of Nursing (Great Britain) : 1987) 2004;18(30):12-14. | Not available |
| Cosgrove L, Flynn C. Marginalized mothers: Parenting without a home. Analyses of Social Issues and Public Policy (ASAP) 2005;5(1):127-43. doi: https://dx.doi.org/10.1111/j.1530-2415.2005.00059.x | Not UK |
| Courtney ME, McMurtry SL, Zinn A. Housing problems experienced by recipients of child welfare services. Child Welfare: Journal of Policy, Practice, and Program 2004;83(5):393-422. | Not UK |
| Cronin-de-Chavez A, Islam S, McEachan RRC. Not a level playing field: A qualitative study exploring structural, community and individual determinants of greenspace use amongst low-income multi-ethnic families. Health & Place 2019;56:118-26. doi: 10.1016/j.healthplace.2019.01.018 | Nothing on HI |
| Cutuli JJ, Herbers JE, Rinaldi M, et al. Asthma and behavior in homeless 4- to 7-year-olds. Pediatrics 2010;125(1):e145-e51. doi: https://dx.doi.org/10.1542/peds.2009-0103 | Not UK |
| D'Amico EJ, Barnes D, Gilbert ML, et al. Developing a tripartite prevention program for impoverished young women transitioning to young adulthood: Addressing substance use, HIV risk, and victimization by intimate partners. Journal of Prevention & Intervention in the Community 2009;37(2):112-28. doi: https://dx.doi.org/10.1080/10852350902735726 | Not UK |
| Daynes L. The health impacts of the refugee crisis: a medical charity perspective. Clinical medicine (London, England) 2016;16(5):437-40. | Essay/narrative review, no UK studies |
| Dickerson A, Popli G. The Many Dimensions of Child Poverty: Evidence from the UK Millennium Cohort Study(*). Fiscal Studies 2018;39(2):265-98. doi: 10.1111/1475-5890.12162 | Not qualitative |
| Donnison D. AFTER CATHY. Community Care. Sutton: Mark Allen Group Ltd, 2007:26-28. | Not research (commentary) |
| Doyle G, Keane E. 'Education comes second to surviving': parental perspectives on their child/ren's early school leaving in an area challenged by marginalisation. Irish Educational Studies 2019;38(1):71-88. doi: 10.1080/03323315.2018.1512888 | Not related to housing insecurity |
| Drennan VM, Joseph J. Health visiting and refugee families: issues in professional practice. Journal of Advanced Nursing 2005;49(2):155-63. doi: 10.1111/j.1365-2648.2004.03282.x | Not related to housing insecurity |
| D'Sa S, Foley D, Hannon J, et al. The psychological impact of childhood homelessness-a literature review. Irish Journal of Medical Science 2021;190(1):411-17. doi: 10.1007/s11845-020-02256-w | Review – refs checked |
| Duck WO. An ethnographic portrait of a precarious life: Getting by on even less. Annals of the American Academy of Political and Social Science 2012;642(1):124-38. doi: https://dx.doi.org/10.1177/0002716212438202 | Not UK |
| Dunleavy A, Kennedy LA, Vaandrager L. Wellbeing for homeless people: a Salutogenic approach. Health promotion international 2014;29(1):144-54. doi: https://dx.doi.org/10.1093/heapro/das045 | Not related to children / families |
| Dyson C, Gorin S, Hooper C-A, et al. Bangladeshi families living in hardship: findings from research using a life-history approach. Child & Family Social Work 2009;14(3):362-71. doi: 10.1111/j.1365-2206.2008.00608.x | Not related to housing insecurity |
| Edidin JP, Ganim Z, Hunter SJ, et al. The mental and physical health of homeless youth: A literature review. Child Psychiatry and Human Development 2012;43(3):354-75. doi: https://dx.doi.org/10.1007/s10578-011-0270-1 | Review – checked refs |
| Edwards R, Weller S, Davidson E, et al. Small Stories of Home Moves: A Gendered and Generational Breadth-and-Depth Investigation. Sociological Research Online 2021 doi: 10.1177/13607804211042033 | Not related to housing insecurity |
| Egan M, Lawson L, Kearns A, et al. Neighbourhood demolition, relocation and health. A qualitative longitudinal study of housing-led urban regeneration in Glasgow, UK. Health & Place 2015;33:101-08. doi: 10.1016/j.healthplace.2015.02.006 | No data on impact of HI on children’s health/wellbeing |
| Evans R. Young Caregiving and HIV in the UK: Caring Relationships and Mobilities in African Migrant Families. Population Space and Place 2011;17(4):338-60. doi: 10.1002/psp.583 | No data on impact of HI on children’s health/wellbeing |
| Finfgeld-Connett D. Becoming homeless, being homeless, and resolving homelessness among women. Issues in mental health nursing 2010;31(7):461-9. doi: https://dx.doi.org/10.3109/01612840903586404 | Review – refs checked |
| Fisher H. Poverty and homelessness. A practice beyond cultural humility: How clinicians can work more effectively in a diverse world 2020:55-60. doi: https://dx.doi.org/10.4324/9780429340901-7 | Not available |
| Fitzpatrick J. QNI Opening Doors project--improving health for homeless people and families. Community practitioner : the journal of the Community Practitioners' & Health Visitors' Association 2012;85(2):19-22. | No data on impact of HI on children’s health/wellbeing |
| Fitzpatrick S, Watts B. Competing visions: security of tenure and the welfarisation of English social housing. Housing Studies 2017;32(8):1021-38. doi: http://dx.doi.org/10.1080/02673037.2017.1291916 | No data on impact of HI on children’s health/wellbeing |
| Flaherty J, Garratt E. Life history mapping: Exploring journeys into and through housing and homelessness. Qualitative Research 2022 doi: 10.1177/14687941211072788 | No data on impact of HI on children’s health/wellbeing |
| Fordham M. The lived experience of homeless women: insights gained as a specialist practitioner. Community practitioner : the journal of the Community Practitioners' & Health Visitors' Association 2015;88(4):32-7. | No data on impact of HI on children’s health/wellbeing |
| Fransham M, Dorling D. Homelessness and public health. BMJ (Online) 2018;360:k214. doi: http://dx.doi.org/10.1136/bmj.k214 | No data on impact of HI on children’s health/wellbeing |
| Gosling VK. 'I've always managed, that's what we do': Social capital and women's experiences of social exclusion. Sociological Research Online 2008;13(1-2) | No data on impact of HI on children’s health/wellbeing |
| Guarino K, Rubin L, Bassuk E. Trauma in the lives of homeless families. Trauma psychology: Issues in violence, disaster, health, and illness, Vol 2: Health and illness 2007:231-58. | eBook not in library, only sample available online |
| Harrington BE, Heyman B, Merleau-Ponty N, et al. Keeping warm and staying well: findings from the qualitative arm of the Warm Homes Project. Health & Social Care in the Community 2005;13(3):259-67. doi: 10.1111/j.1365-2524.2005.00558.x | Nothing on HI |
| Harris E, Nowicki M, Brickell K. On-edge in the impasse: Inhabiting the housing crisis as structure-of-feeling. Geoforum 2019;101:156-64. doi: 10.1016/j.geoforum.2018.09.001 | No data on impact of HI on children’s health/wellbeing |
| Harvey K. "When I go to bed hungry and sleep, I'm not hungry": Children and parents' experiences of food insecurity. Appetite 2016;99:235-44. doi: https://dx.doi.org/10.1016/j.appet.2016.01.004 | Not related to housing insecurity |
| Hawkins RL. Fickle families and the kindness of strangers: Social capital in the lives of low-income single mothers. Journal of Human Behavior in the Social Environment 2010;20(1):38-55. doi: https://dx.doi.org/10.1080/10911350903183263 | Not UK |
| Haynes J, Parsons T. Families in transit. Community practitioner : the journal of the Community Practitioners' & Health Visitors' Association 2009;82(9):16. | No qualitative data |
| Hayter AKM, Draper AK, Ohly HR, et al. A qualitative study exploring parental accounts of feeding pre-school children in two low-income populations in the UK. Maternal and Child Nutrition 2015;11(3):371-84. doi: 10.1111/mcn.12017 | Not related to housing insecurity |
| Hernandez D. Affording housing at the expense of health: Exploring the housing and neighborhood strategies of poor families. Journal of Family Issues 2016;37(7):921-46. doi: https://dx.doi.org/10.1177/0192513X14530970 | Not UK |
| Hickman P, Kemp PA, Reeve K, et al. The impact of the direct payment of housing benefit: evidence from Great Britain. Housing Studies 2017;32(8):1105-26. doi: http://dx.doi.org/10.1080/02673037.2017.1301401 | No data on impact of HI on children’s health/wellbeing |
| Jolly A, Singh J, Lobo S. No recourse to public funds: a qualitative evidence synthesis. International Journal of Migration Health and Social Care 2022;18(1):107-23. doi: 10.1108/ijmhsc-11-2021-0107 | Review – checked refs |
| Jones D, Lowe P, West K. Austerity in a disadvantaged West Midlands neighbourhood: Everyday experiences of families and family support professionals. Critical Social Policy 2020;40(3):389-409. doi: 10.1177/0261018319840923 | No data on impact of HI on children’s health/wellbeing |
| Khanom A, Hill RA, Brophy S, et al. Mothers' perspectives on the delivery of childhood injury messages: a qualitative study from the growing up in Wales, environments for healthy living study (EHL). Bmc Public Health 2013;13 doi: 10.1186/1471-2458-13-806 | Nothing on HI |
| Knowles M, Rabinowich J, Ettinger de Cuba S, et al. "Do you wanna breathe or eat?": Parent perspectives on child health consequences of food insecurity, trade-offs, and toxic stress. Maternal and Child Health Journal 2016;20(1):25-32. doi: https://dx.doi.org/10.1007/s10995-015-1797-8 | Not UK |
| Koplan C, Chard A. Adverse early life experiences as a social determinant of mental health. Psychiatric Annals 2014;44(1):39-45. doi: https://dx.doi.org/10.3928/00485713-20140108-07 | Not UK |
| Kristiansen IL. Consequences of serious parental health events on child mental health and educational outcomes. Health Economics (United Kingdom) 2021;30(8):1772-817. doi: https://dx.doi.org/10.1002/hec.4278 | Not UK |
| Lorelle S, Grothaus T. Homeless children and their families' perspectives of agency services. Community Mental Health Journal 2015;51(7):800-08. doi: https://dx.doi.org/10.1007/s10597-015-9827-y | Not UK |
| Make or Break: How Homeless Young People Struggle To Fulfil Their Potential. 2001:1-13. | Not available. (Looks unlikely to contain data from the abstract.) |
| Mallett S, Rosenthal D, Keys D. Young people, drug use and family conflict: pathways into homelessness. Journal of adolescence 2005;28(2):185-99. | Not UK |
| Martin D, Sweeney J, Visitor H, et al. Views of teenage parents on their support housing needs. Community practitioner : the journal of the Community Practitioners' & Health Visitors' Association 2005;78(11):392-6. | No data on impact of HI on children’s health/wellbeing |
| Martin-West S. The role of social support as a moderator of housing instability in single mother and two-parent households. Social Work Research 2019;43(1):31-42. doi: https://dx.doi.org/10.1093/swr/svy028 | Not UK |
| Matthews P, Poyner C, Kjellgren R. Lesbian, gay, bisexual, transgender and queer experiences of homelessness and identity: insecurity and home(o)normativity. International Journal of Housing Policy 2019;19(2):232-53. doi: 10.1080/19491247.2018.1519341 | Not related to children / families |
| Mayberry LS, Shinn M, Benton JG, et al. Families experiencing housing instability: The effects of housing programs on family routines and rituals. American Journal of Orthopsychiatry 2014;84(1):95-109. doi: https://dx.doi.org/10.1037/h0098946 | Not UK |
| Mc Conalogue D, Maunder N, Areington A, et al. Homeless people and health: a qualitative enquiry into their practices and perceptions. Journal of Public Health 2021;43(2):287-94. doi: 10.1093/pubmed/fdz104 | Not related to children / families |
| McCabe E, O'Connor J. Home remembered, relived and revised: a qualitative study exploring the experiences of home for homeless persons in supported accommodation. European Journal of Psychotherapy & Counselling 2016;18(3):290-303. doi: 10.1080/13642537.2016.1214162 | Not related to children / families  (Also this may not be UK, unclear) |
| McCann E, Brown M. Homelessness among youth who identify as LGBTQ+: A systematic review. Journal of clinical nursing 2019;28(11-12):2061-72. doi: https://dx.doi.org/10.1111/jocn.14818 | Review – references checked (studies reviewed were non-UK and related to homeless youth not in families) |
| McLoughlin PJ. Couch surfing on the margins: the reliance on temporary living arrangements as a form of homelessness amongst school-aged home leavers. Journal of Youth Studies 2013;16(4):521-45. doi: 10.1080/13676261.2012.725839 | Not UK |
| Menke EM. Comparison of the stressors and coping behaviors of homeless, previously homeless, and never homeless poor children. Issues in Mental Health Nursing 2000;21(7):691-710. doi: https://dx.doi.org/10.1080/01612840050207617 | Not UK |
| Mercer SW, Cawston PG, Bikker AP. Quality in general practice consultations; a qualitative study of the views of patients living in an area of high socio-economic deprivation in Scotland. Bmc Family Practice 2007;8 doi: 10.1186/1471-2296-8-22 | Not related to housing at all |
| Merrick MT, Henly M, Turner HA, et al. Beyond residential mobility: A broader conceptualization of instability and its impact on victimization risk among children. Child Abuse & Neglect 2018;79:485-94. doi: https://dx.doi.org/10.1016/j.chiabu.2018.01.029 | Not UK |
| Meschede T, Chaganti S. Home for now: A mixed-methods evaluation of a short-term housing support program for homeless families. Evaluation and Program Planning 2015;52:85-95. doi: https://dx.doi.org/10.1016/j.evalprogplan.2015.03.009 | Not UK |
| Miller PM, Pavlakis A, Samartino L, et al. Brokering educational opportunity for homeless students and their families. International Journal of Qualitative Studies in Education 2015;28(6):730-49. doi: https://dx.doi.org/10.1080/09518398.2015.1017860 | Not UK |
| Miller PM. A critical analysis of the research on student homelessness. Review of Educational Research 2011;81(3):308-37. doi: https://dx.doi.org/10.3102/0034654311415120 | Review – Checked refs |
| Miller PM. Families' experiences in different homeless and highly mobile settings: Implications for school and community practice. Education and Urban Society 2015;47(1):3-32. doi: https://dx.doi.org/10.1177/0013124512469814 | Not UK |
| Morris RI, Strong L. The Impact of Homelessness on the Health of Families. The Journal of School Nursing 2004;20(4):221-27. doi: https://dx.doi.org/10.1622/1059-8405%282004%29020%5B0221:TIOHOT%5D2.0.CO;2 | Not UK |
| Mulrenan P, Atkins J, Cox S. "I get up in the night to cry': The impact of homelessness on higher education students in London, UK. Critical Social Policy 2018;38(1):143-54. doi: 10.1177/0261018317724524 | No data on impact of HI on children’s health/wellbeing |
| Mulrenan P, Atkins J, Cox S. 'I didn't know what strong was until it was required': factors that promote retention among homeless students in higher education. Journal of Further and Higher Education 2020;44(2):273-84. doi: 10.1080/0309877x.2018.1533929 | No data on impact of HI on children’s health/wellbeing |
| Murray ET, Lacey R, Maughan B, et al. Association of childhood out-of-home care status with all-cause mortality up to 42-years later: Office of National Statistics Longitudinal Study. Bmc Public Health 2020;20(1) doi: 10.1186/s12889-020-08867-3 | Not qualitative |
| Nabors LA, Weist MD, Shugarman R, et al. Assessment, Prevention, and Intervention Activities in a School-Based Program for Children Experiencing Homelessness. Special Issue: Expanded School Mental Health: Exploring Program Details and Developing the Research Base 2004;28(4):565-78. doi: https://dx.doi.org/10.1177/0145445503259517 | Not UK |
| Nambi B, Majumder P, Vostanis P. Relationship of psychosocial adversity to depressive symptoms and self-harm in young homeless people. International psychiatry : bulletin of the Board of International Affairs of the Royal College of Psychiatrists 2012;9(2):40-42. | Not qualitative |
| Naven L, Egan J, Sosu EM, et al. The influence of poverty on children's school experiences: pupils' perspectives. Journal of Poverty and Social Justice 2019;27(3):313-31. doi: 10.1332/175982719x15622547838659 | No mention of housing |
| Nwokah EE, Becerril S, Hardee WP, et al. Play with homeless and low-income preschoolers: University student experiences with service learning. International Journal of Play 2017;6(1):53-77. doi: https://dx.doi.org/10.1080/21594937.2017.1288397 | Not UK |
| O'Brien N, Joyce B, Hayes AM, et al. Parental perceptions regarding the impact of housing on health. Archives of Disease in Childhood 2021;106(SUPPL 1):A250. doi: http://dx.doi.org/10.1136/archdischild-2021-rcpch.434 | Conference abstract, not qualitative, not UK |
| O'Reilly M, Taylor HC, Vostanis P. "Nuts, schiz, psycho": An exploration of young homeless people's perceptions and dilemmas of defining mental health. Social Science & Medicine 2009;68(9):1737-44. doi: 10.1016/j.socscimed.2009.02.033 | No data on impact of HI on children’s health/wellbeing |
| Osypuk TL, Schmidt N, Nguyen Q, et al. Effects of housing mobility and lower poverty neighborhoods on adolescent asthma: The moving to opportunity experiment. American Journal of Epidemiology 2012;175(SUPPL. 11):S48. doi: http://dx.doi.org/10.1093/aje/kws258 | Conference abstract, not qualitative |
| Park JM, Fertig AR, Allison PD. Physical and mental health, cognitive development, and health care use by housing status of low-income young children in 20 American cities: A prospective cohort study. American Journal of Public Health 2011;101(Suppl 1):S255-S61. doi: https://dx.doi.org/10.2105/AJPH.2010.300098 | Not UK |
| Parks RW, Stevens RJ, Spence SA. A systematic review of cognition in homeless children and adolescents. Journal of the Royal Society of Medicine 2007;100(1):46-50. doi: http://dx.doi.org/10.1258/jrsm.100.1.46 | Review – not relevant as quantitative studies and none were UK |
| Parr S. Family Policy and the Governance of Anti-Social Behaviour in the UK: Women's Experiences of Intensive Family Support. Journal of Social Policy 2011;40:717-37. doi: 10.1017/s0047279410000735 | Impact on mothers, not children. Not health outcomes. |
| Parry BJ, Quinton ML, Holland MJG, et al. Improving outcomes in young people experiencing homelessness with My Strengths Training for Life (TM) (MST4Life (TM)): A qualitative realist evaluation. Children and Youth Services Review 2021;121 doi: 10.1016/j.childyouth.2020.105793 | Population (youth not in family) |
| Parry BJ, Thompson JL, Holland MJG, et al. Promoting Personal Growth in Young People Experiencing Homelessness Through an Outdoors-Based Program. Journal of Youth Development 2021;16(5):157-92. doi: 10.5195/jyd.2021.1061 | Population (youth not in family) |
| Parry Y, Willis E, Kendall S, et al. A Nurse Practitioner service designed to address the health needs of children living in housing instability: A non-randomised, concurrent mixed methods study protocol. Journal of advanced nursing 2022;78(4):1166-75. doi: https://dx.doi.org/10.1111/jan.15152 | Study protocol |
| Pasqualini M, Lanari D, Pieroni L. Parents who exit and parents who enter. Family structure transitions, child psychological health, and early drinking. Social science & medicine (1982) 2018;214:187-96. doi: https://dx.doi.org/10.1016/j.socscimed.2018.08.017 | Family instability (e.g. parent leaving home), not housing insecurity. |
| Pates RM, Hooper K. Drug use and mental health in a Secure Children's Home. Advances in Dual Diagnosis 2017;10(2):71-82. doi: 10.1108/add-09-2016-0016 | Population (youth not in family) |
| Paton J, Crouch W, Camic P. Young offenders' experiences of traumatic life events: a qualitative investigation. Clinical child psychology and psychiatry 2009;14(1):43-62. doi: https://dx.doi.org/10.1177/1359104508100135 | Extracted LB |
| Pavis S, Hubbard G, Platt S. Young people in rural areas: Socially excluded or not? Work Employment and Society 2001;15(2):291-309. doi: 10.1177/09500170122118968 | Rurality, employment opportunities and social exclusion – not housing insecurity. |
| Pavlakis AE, Goff P, Miller PM. Contextualizing the impacts of homelessness on academic growth. Teachers College Record 2017;119(10):1-23. | Not UK |
| Pavlakis AE. Reaching all families: Family, school, and community partnerships amid homelessness and high mobility in an urban district. Urban Education 2018;53(8):1043-73. doi: https://dx.doi.org/10.1177/0042085915613547 | Not UK |
| Perlman S, Cowan B, Gewirtz A, et al. Promoting Positive Parenting in the Context of Homelessness. American Journal of Orthopsychiatry 2012;82(3):402-12. doi: http://dx.doi.org/10.1111/j.1939-0025.2012.01158.x | Non-UK (USA) |
| Perry TE, Hassevoort L, Petrusak J. Care networks in play: Understanding death of a parent as a contributing factor to homelessness. Journal of Human Behavior in the Social Environment 2017;27(7):656-68. doi: https://dx.doi.org/10.1080/10911359.2017.1319316 | Not UK |
| Pevalin DJ, Taylor MP, Todd J. The dynamics of unhealthy housing in the UK: A panel data analysis. Housing Studies 2008;23(5):679-95. doi: 10.1080/02673030802253848 | Data on adults not children. |
| Phinney R. Exploring residential mobility among low-income families. Social Service Review 2013;87(4):780-815. doi: https://dx.doi.org/10.1086/673963 | Not UK |
| Plewis I, Smith G, Wright G, et al. Linking Child Poverty and Child Outcomes: Exploring Data and Research Strategies. Research Working Paper: Social Research Branch, Department for Work and Pensions, 4th Floor, Adelphi, 1-11 John Adam St., London WC2N 6HT England. For full text: http://www.dwp.gov.uk/asd/asd5/WP1.pdf, 2001:1-92. | Poverty and household income – not insecurity. |
| Pluck G, Lee K-H, David R, et al. Neurobehavioural and cognitive function is linked to childhood trauma in homeless adults. British Journal of Clinical Psychology 2011;50:33-45. doi: 10.1348/014466510x490253 | Emotional/physical neglect in childhood – not housing insecurity. |
| Poulter H, Eberhardt J, Moore H, et al. "Bottom of the Pile": Health Behaviors within the Context of In-work Poverty in North East England. Journal of Poverty 2022 doi: 10.1080/10875549.2021.2023721 | No mention of housing |
| Poulter H, Eberhardt J, Moore H, et al. 'Absorbing the shock': Food scarcity and eating behaviours within the context of in-work poverty in North East England. Obesity Reviews 2020;21(SUPPL 1) doi: https://dx.doi.org/10.1111/obr.13118 | Conference abstract – no mention of housing |
| Powdthavee N, Vernoit J. Parental unemployment and children's happiness: A longitudinal study of young people's well-being in unemployed households. Labour economics 2013;24:253-63. | Household income and unemployment – not housing insecurity. |
| Power L, Raphael D. Care leavers: A British affair. Child & Family Social Work 2018;23(3):346-53. doi: https://dx.doi.org/10.1111/cfs.12421 | Population (youth not in family) |
| Price C, Dalman C, Zammit S, et al. Association of Residential Mobility Over the Life Course With Nonaffective Psychosis in 1.4 Million Young People in Sweden. JAMA psychiatry 2018;75(11):1128-36. doi: https://dx.doi.org/10.1001/jamapsychiatry.2018.2233 | Non-UK (Sweden) |
| Raffaele Mendez LM, Randle CA. Lifted: A thematic analysis of homeless adolescents' reflections on their lives since beginning a multifaceted, community-based intervention. Children and Youth Services Review 2021;121 doi: https://dx.doi.org/10.1016/j.childyouth.2020.105891 | Not UK |
| Reading R, Steel S, Reynolds S. Citizens advice in primary care for families with young children. Child Care Health and Development 2002;28(1):39-45. doi: 10.1046/j.1365-2214.2002.00241.x | No mention of housing |
| Rees G, Bradshaw J. Exploring Low Subjective Well-Being Among Children Aged 11 in the UK: an Analysis Using Data Reported by Parents and by Children. Child Indicators Research 2018;11(1):27-56. doi: 10.1007/s12187-016-9421-z | Housing tenure and quality – not insecurity |
| Rees G. Variations in Children's Affective Subjective Well-Being at Seven Years Old: an Analysis of Current and Historical Factors. Child Indicators Research 2019;12(1):141-60. doi: 10.1007/s12187-017-9516-1 | Incoming and housing tenure (not insecurity) |
| Reid S, Berman H, Forchuk C. Living on the streets in Canada: A feminist narrative study of girls and young women. Issues in Comprehensive Pediatric Nursing 2005;28(4):237-56. doi: https://dx.doi.org/10.1080/01460860500396906 | Not UK |
| Richards J, Kliner M, Brierley S, et al. Maternal and infant health of Eastern Europeans in Bradford, UK: a qualitative study. Community practitioner : the journal of the Community Practitioners' & Health Visitors' Association 2014;87(9):33-6. | No mention of housing |
| Ridge T. It's a family affair: Low-income children's perspectives on maternal work. Journal of Social Policy 2007;36:399-416. doi: 10.1017/s0047279407001109 | Lone parent households and income – but not housing insecurity |
| Riley R, Johnson T, Pearson L. An audit into homeless families in temporary accommodation. British journal of community nursing 2001;6(1):18-25. | Lack of detail, little qualitative findings, no data |
| Ritchie C, Buchanan A. Self-Report of Parenting Style, Socio-economic Status and Psychological Functioning in a Community Sample of 13-15-year-old Students. Journal of Social Work 2010;10(3):317-32. doi: 10.1177/1468017310361857 | Parenting style and wellbeing. No mention of housing insecurity. |
| Rivera L. Changing women: An ethnographic study of homeless mothers and popular education. Journal of Sociology and Social Welfare 2003;30(2):31-51. | Not UK |
| Robb C. For whose benefit? Public Finance 2010:12. | Not research (commentary) |
| Roche J, Tucker S. Extending the social exclusion debate: An exploration of the family lives of young carers and young people with ME. Childhood: A Global Journal of Child Research 2003;10(4):439-56. doi: https://dx.doi.org/10.1177/0907568203104004 | Impact of having ME on young people. |
| Rolfe S, Garnham L, Godwin J, et al. Housing as a social determinant of health and wellbeing: developing an empirically-informed realist theoretical framework. Bmc Public Health 2020;20(1) doi: 10.1186/s12889-020-09224-0 | Data on adults, not children. |
| Rollins JH, Saris RN, Johnston-Robledo I. Low-income women speak out about housing: A high-stakes game of musical chairs. Special Issue: Listening to the Voices of Poor Women 2001;57(2):277-98. doi: https://dx.doi.org/10.1111/0022-4537.00213 | Not UK |
| Rose W, McAuley C. Poverty and its impact on parenting in the UK: Re-defining the critical nature of the relationship through examining lived experiences in times of austerity. Children and Youth Services Review 2019;97:134-41. doi: 10.1016/j.childyouth.2017.10.021 | Mentions housing quality but not insecurity. |
| Rose-Jacobs R, Ettinger de Cuba S, Bovell-Ammon A, et al. Housing Instability Among Families With Young Children With Special Health Care Needs. Pediatrics 2019;144(2) doi: https://dx.doi.org/10.1542/peds.2018-1704 | Not UK |
| Rosenthal D, Heys M, Schoenthaler A, et al. 920 Identifying housing-level barriers to optimal health for Under5s experiencing homelessness: a citizen science approach. Archives of Disease in Childhood 2021;106(Suppl 1):A163. doi: http://dx.doi.org/10.1136/archdischild-2021-rcpch.283 | Conference abstract, inadequate detail |
| Roze M, Vandentorren S, Vuillermoz C, et al. Emotional and behavioral difficulties in children growing up homeless in Paris. Results of the ENFAMS survey. European Psychiatry 2016;38:51-60. doi: https://dx.doi.org/10.1016/j.eurpsy.2016.05.001 | Not available |
| Sandel M, Sheward R, de Cuba SE, et al. Unstable housing and caregiver and child health in renter families. Pediatrics 2018;141(2):1-10. doi: https://dx.doi.org/10.1542/peds.2017-2199 | Not UK |
| Sarkar K, Rosenthal DM, Martinez DC, et al. Mitigating the severity of child homelessness in the UK: a global mixed-methods systematic review. The Lancet 2021;398(Supplement 2):S75. doi: https://dx.doi.org/10.1016/S0140-6736%2821%2902618-0 | Review of low and middle-income countries (also conference abstract) |
| Satherley R-M, Wolfe I, Lingam R. Experiences of healthcare for mothers of children with ongoing illness, living in deprived neighbourhoods health and place. Health & place 2021;71:102661. doi: https://dx.doi.org/10.1016/j.healthplace.2021.102661 | Not housing insecurity |
| Schindler H. S., Coley R. L. A Qualitative Study of Homeless Fathers: Exploring Parenting and Gender Role Transitions, Family Relations: An Interdisciplinary Journal of Applied Family Studies 2007: 56: 40-51. | Not UK |
| Schmitz CL, Wagner JD, Menke EM. The interconnection of childhood poverty and homelessness: Negative impact/points of access. Families in Society 2001;82(1):69-77. doi: https://dx.doi.org/10.1606/1044-3894.223 | Not UK |
| Schoon I, Jones E, Cheng H, et al. Family hardship, family instability, and cognitive development. Journal of Epidemiology and Community Health 2012;66(8):716-22. doi: 10.1136/jech.2010.121228 | Not qualitative |
| Shortt NK, Ross C. Children's perceptions of environment and health in two Scottish neighbourhoods. Social Science & Medicine 2021;283 doi: 10.1016/j.socscimed.2021.114186 | No mention of housing |
| Silva MR, Kleinert WL, Sheppard AV, et al. The relationship between food security, housing stability, and school performance among college students in an urban university. Journal of College Student Retention: Research, Theory and Practice 2017;19(3):284-99. doi: https://dx.doi.org/10.1177/1521025115621918 | Not UK |
| Simon A. Early access and use of housing: care leavers and other young people in difficulty. Child & Family Social Work 2008;13(1):91-100. doi: 10.1111/j.1365-2206.2007.00524.x | Set of abstracts with references – none relevant |
| Smith D, Roberts R. Young parents: the role of housing in understanding social inequality. The journal of family health care 2011;21(1):20-2. | Teenage pregnancy |
| Stanistreet P. "We're like a Family". Adults Learning 2008;19(5):14-16. | Not available |
| Stevenson C, Wakefield JRH, Kellezi B, et al. Families as support and burden: A mixed methods exploration of the extent to which family identification and support predicts reductions in stress among disadvantaged neighbourhood residents. Journal of Social and Personal Relationships 2022;39(4):886-907. doi: 10.1177/02654075211050071 | Not qualitative |
| Stewart J, Rhoden M. Children, housing and health. The International Journal of Sociology and Social Policy 2006;26(7/8):326-41. doi: http://dx.doi.org/10.1108/01443330610680416 | Review, references checked |
| Sturm DC, Hill C. Play therapy with children experiencing homelessness. [Play therapy: A comprehensive guide to theory and practice](https://ebookcentral.proquest.com/lib/sheffield/detail.action?docID=1760717&pq-origsite=primo) 2015:276-89. | Ebook – available online. Nothing of relevance. |
| Sylvestre J, Kerman N, Polillo A, et al. A qualitative study of the pathways into and impacts of family homelessness. Journal of Family Issues 2018;39(8):2265-85. doi: https://dx.doi.org/10.1177/0192513X17746709 | Not UK |
| Tach L, Dunifon R, Miller DL. Confronting inequality: How policies and practices shape children's opportunities. Confronting inequality: How policies and practices shape children's opportunities 2020 doi: https://dx.doi.org/10.1037/0000187-000 | Book – not available |
| Tach LM, Greene SS. "Robbing Peter to pay Paul": Economic and cultural explanations for how lower-income families manage debt. Social Problems 2014;61(1):1-21. doi: https://dx.doi.org/10.1525/sp.2013.11262 | Not UK |
| Tam CC, Freisthler B, Curry SR, et al. Where are the beds? Housing locations for transition age youth exiting public systems. Families in Society 2016;97(2):111-19. doi: https://dx.doi.org/10.1606/1044-3894.2016.97.12 | Not UK |
| Tarasuk V, Cheng J, de Oliveira C, et al. Association between household food insecurity and annual health care costs. Canadian Medical Association Journal 2015;187(14):E429-E36. doi: https://dx.doi.org/10.1503/cmaj.150234 | Not UK |
| Thompson M, Hooper C, Laver-Bradbury C, et al. Child and adolescent mental health: Theory and practice. [Child and adolescent mental health: Theory and practice](https://www.proquest.com/docview/2510624173/364C45B3E402470APQ/1?accountid=13828) 2012 | Book – available online. Nothing of relevance, checked refs |
| Topp L, Hudson SL, Maher L. Mental health symptoms among street-based psychostimulant injectors in Sydney's Kings Cross. Substance Use & Misuse 2010;45(7-8):1180-200. doi: https://dx.doi.org/10.3109/10826080903443586 | Not UK |
| Torchalla I, Linden IA, Strehlau V, et al. "Like a lots happened with my whole childhood": Violence, trauma, and addiction in pregnant and postpartum women from Vancouver's Downtown Eastside. Harm Reduction Journal 2015;11 | Not UK |
| Torchalla I, Linden IA, Strehlau V, et al. "Like a lots happened with my whole childhood": Violence, trauma, and addiction in pregnant and postpartum women from Vancouver's Downtown Eastside": Erratum. Harm Reduction Journal 2017;14 doi: https://dx.doi.org/10.1186/s12954-017-0191-9 | Erratum to an excluded article |
| Trapp CM, Burke G, Gorin AA, et al. The relationship between dietary patterns, body mass index percentile, and household food security in young urban children. Childhood Obesity 2015;11(2):148-55. doi: https://dx.doi.org/10.1089/chi.2014.0105 | Not available – but does not look relevant (looks like US and quantitative) |
| Trevena P, McGhee D, Heath S. Location, Location? A Critical Examination of Patterns and Determinants of Internal Mobility Among Post-accession Polish Migrants in the UK. Population Space and Place 2013;19(6):671-87. doi: 10.1002/psp.1788 | No reference to impact on child health/wellbeing |
| Trofholz AC, Tate A, Keithahn H, et al. Family meal characteristics in racially/ethnically diverse and immigrant/refugee households by household food security status: A mixed methods study. Appetite 2021;157 doi: https://dx.doi.org/10.1016/j.appet.2020.105000 | Not UK |
| Tseliou F, Maguire A, Donnelly M, et al. The influence of mobility on mental health status in young people: The role of area-level deprivation. Health & Place 2016;42:96. | Not qualitative |
| Tseng KK, Park SH, Shearston JA, et al. Parental psychological distress and family food insecurity: Sad dads in hungry homes. Journal of Developmental and Behavioral Pediatrics 2017;38(8):611-18. doi: https://dx.doi.org/10.1097/DBP.0000000000000481 | Not available – but does not look relevant (looks like US) |
| Tshabangu I, Tshabangu T. LONE PARENTING: A STUDY OF LONE MOTHERS IN THE ERA OF FISCAL AUSTERITY. 8th International Conference of Education, Research and Innovation (ICERI); 2015 2015 Nov 16-20; Seville, SPAIN. | Full text not available – conference abstract, insufficient detail |
| Turnbull H, Loptson K, Muhajarine N. Experiences of housing insecurity among participants of an early childhood intervention programme. Child: Care, Health and Development 2014;40(3):435-40. doi: https://dx.doi.org/10.1111/cch.12091 | Not UK |
| Turney K, Harknett K. Neighborhood disadvantage, residential stability, and perceptions of instrumental support among new mothers. Journal of Family Issues 2010;31(4):499-524. doi: https://dx.doi.org/10.1177/0192513X09347992 | Not UK |
| van Kranenburg GD, Diekman WJ, Mulder WG, et al. Histories of social functioning and mental healthcare in severely dysfunctional dual-diagnosis psychiatric patients. International Journal of Mental Health and Addiction 2020;18(4):904-16. doi: https://dx.doi.org/10.1007/s11469-018-9992-7 | Not UK |
| Veeran V. Working With Street Children: A Child-centred Approach. Child Care in Practice 2004;10(4):359-66. doi: https://dx.doi.org/10.1080/1357527042000285538 | Lone children – not families |
| Velonis AJ, Daoud N, Matheson F, et al. Strategizing safety: Theoretical frameworks to understand women's decision making in the face of partner violence and social inequities. Journal of Interpersonal Violence 2017;32(21):3321-45. doi: https://dx.doi.org/10.1177/0886260515598953 | Not UK |
| Ventriglio A, Mari M, Bellomo A, et al. Homelessness and mental health: A challenge. International Journal of Social Psychiatry 2015;61(7):621-22. doi: https://dx.doi.org/10.1177/0020764015585680 | (Narrative) review – checked refs |
| Vernon-Feagans L, Garrett-Peters P, Willoughby M, et al. Chaos, poverty, and parenting: Predictors of early language development. Early Childhood Research Quarterly 2012;27(3):339-51. doi: https://dx.doi.org/10.1016/j.ecresq.2011.11.001 | Not UK |
| Vollmann WT. Poor people. Poor people 2007 | Not available (but does not look relevant – no mention of UK but other countries) |
| Vostanis P, Tischler V, Cumella S, et al. Mental health problems and social supports among homeless mothers and children victims of domestic and community violence. International Journal of Social Psychiatry 2001;47(4):30-40. doi: 10.1177/002076400104700403 | No qualitative data reported |
| Vostanis P. Mental health of homeless children and their families. Advances in Psychiatric Treatment 2002;8(6):463-69. doi: http://dx.doi.org/10.1192/apt.8.6.463 | No data from children / parents included. Just fictitious case studies. |
| Wager F, Hill M, Bailey N, et al. The Impact of Poverty on Children and Young People's Use of Services. Children & Society 2010;24(5):400-12. doi: 10.1111/j.1099-0860.2009.00236.x | Nothing re. (perceived/experience of) housing insecurity |
| Walker B, Niner P. Welfare or Work? Low-Income Working Households' Housing Consumption in the Private Rented Sector in England. Housing Studies 2012;27(3):381-97. doi: 10.1080/02673037.2012.651108 | Nothing re children’s health/wellbeing |
| Walsh E. "Family-friendly" tenancies in the private rented sector. Journal of Property Planning and Environmental Law 2019;11(3):230-43. doi: 10.1108/jppel-04-2019-0020 | No data, no reference to a relevant UK study |
| Ward K, Fagan C, McDowell L, et al. Living and working in urban working class communities. Geoforum 2007;38(2):312-25. doi: 10.1016/j.geoforum.2006.05.003 | Nothing re. housing instability impact on children |
| Warren EJ, Font SA. Housing insecurity, maternal stress, and child maltreatment: An application of the family stress model. Social Service Review 2015;89(1):9-39. doi: https://dx.doi.org/10.1086/680043 | Not UK |
| Watson J, Crawley J, Kane D. Social exclusion, health and hidden homelessness. Public Health 2016;139:96-102. doi: https://dx.doi.org/10.1016/j.puhe.2016.05.017 | Not UK |
| Wehler C, Weinreb LF, Huntington N, et al. Risk and Protective Factors for Adult and Child Hunger Among Low-income Housed and Homeless Female-Headed Families. American Journal of Public Health 2004;94(1):109-15. doi: https://dx.doi.org/10.2105/AJPH.94.1.109 | Not UK |
| Weis L, Marcus W, Freie C. Living with violence: Housed and homeless women and children. Invisible children in the society and its schools, 2nd ed 2003:193-215. | Book, not available online |
| Wells NM. Our housing, our selves: A longitudinal investigation of low-income women's participatory housing experiences. Journal of Environmental Psychology 2005;25(2):189-206. doi: https://dx.doi.org/10.1016/j.jenvp.2005.02.002 | Not UK |
| Wells-Wilbon R, Jones K, Rich T. [Mental and emotional wellness among African Americans in urban environments: What do we know? How can we improve outcomes? Social work practice with African Americans in urban environments](https://ebookcentral.proquest.com/lib/sheffield/detail.action?docID=4096379&pq-origsite=primo) 2016:207-24. | Not UK, not much on housing |
| Wenzel SL, Hambarsoomian K, D'Amico EJ, et al. Victimization and health among indigent young women in the transition to adulthood: A portrait of need. Journal of Adolescent Health 2006;38(5):536-43. doi: https://dx.doi.org/10.1016/j.jadohealth.2005.03.019 | Not UK |
| Wershler JL, Ronis ST. Psychosocial characteristics and service needs of Canadian suburban male youth at risk for homelessness. Children and Youth Services Review 2015;55:29-36. doi: https://dx.doi.org/10.1016/j.childyouth.2015.05.009 | Not UK |
| Western B, Braga AA, Davis J, et al. Stress and hardship after prison. American Journal of Sociology 2015;120(5):1512-47. doi: https://dx.doi.org/10.1086/681301 | Not UK |
| Wildman JM. "It's Luck as to What Sort of Family You're Born Into" Cumulative Dis/advantage Generative Systemic Processes Across the Life Course of a Baby-Boom Birth Cohort. Journals of Gerontology Series B-Psychological Sciences and Social Sciences 2020;75(6):1302-11. doi: 10.1093/geronb/gbaa017 | Nothing re. health / wellbeing impact on children.  This study’s interviews were conducted when the cohort was aged 67–68. |
| Williams S, Stickley T. Stories from the streets: people's experiences of homelessness. Journal of Psychiatric and Mental Health Nursing 2011;18(5):432-39. doi: 10.1111/j.1365-2850.2010.01676.x | Nothing re. children |
| Wingate-Lewinson T, Hopps JG, Reeves P. Liminal living at an extended stay hotel: Feeling "stuck" in a housing solution. Journal of Sociology and Social Welfare 2010;37(2):9-34. | Not UK |
| Wood H. When only a house makes a home: How home selection matters in the residential mobility decisions of lower-income, inner-city African American families. Social Service Review 2014;88(2):264-94. doi: https://dx.doi.org/10.1086/676407 | Not UK |
| Workman CL, Ureksoy H. Water insecurity in a syndemic context: Understanding the psycho-emotional stress of water insecurity in Lesotho, Africa. Social Science & Medicine 2017;179:52-60. doi: https://dx.doi.org/10.1016/j.socscimed.2017.02.026 | Not UK |
| Wu C-F, Eamon MK. Does receipt of public benefits reduce material hardship in low-income families with children? Children and Youth Services Review 2010;32(10):1262-70. doi: https://dx.doi.org/10.1016/j.childyouth.2010.04.017 | Not UK |
| Xu X, Zhu X, Bresnahan M. Fighting back: Inner-city community responses to food insecurity. American Behavioral Scientist 2016;60(11):1306-21. doi: https://dx.doi.org/10.1177/0002764216657380 | Not UK |
| Yeager KR, Minkoff K. Establishing a comprehensive, continuous, integrated system of care for persons with co-occurring conditions. Modern community mental health: An interdisciplinary approach 2013:497-515. | Book chapter, no detail on housing |
| Yuen FKO. Social work practice with children and families: A family health approach. [Social work practice with children and families: A family health approach](https://ebookcentral.proquest.com/lib/sheffield/detail.action?pq-origsite=primo&docID=668660) 2005 | Book available online as an ebook – nothing of relevance |
| Zufferey C. Homelessness, social policy, and social work: A way forward. Australian Social Work 2011;64(3):241-44. doi: https://dx.doi.org/10.1080/0312407X.2011.607770 | Editorial (also not UK) |
