# Supplementary material for: Exploring the impact of housing insecurity on the health and wellbeing of children and young people in the United Kingdom: a qualitative systematic review
Source: BMC Public Health. 2024 Sep 9;24:2453. doi: 10.1186/s12889-024-19735-9 (PMC11385840; doi:10.1186/s12889-024-19735-9)
Supplement: Supplementary file 4 — Supplementary Material 4. [file 12889_2024_19735_MOESM4_ESM.docx]

## **Exploring the impact of housing insecurity on the health and wellbeing of children and young people in the United Kingdom: a qualitative systematic review**

## **Additional File 4: Quality appraisal of included studies**

File name: Additional File 4 - Quality appraisal of tables

File type: Microsoft Word Document (.docx)

Title of data: Supplementary Table 3: Quality appraisal of published literature using the Critical Appraisal Skills Programme (CASP) qualitative checklist;

Supplementary Table 3: Quality appraisal of grey literature using the Authority, Accuracy, Coverage, Objectivity, Date, Significance (AACODS) checklist

Description of data: Quality appraisal of published and grey literature using the Critical Appraisal Skills Programme (CASP) qualitative checklist and the Authority, Accuracy, Coverage, Objectivity, Date, Significance (AACODS) checklist, respectively. For each source document, both ratings and a summary have been presented.

**Supplementary Table 3: Quality appraisal of published literature using the Critical Appraisal Skills Programme (CASP) qualitative checklist**

| **First author / year** | **Design** | **N=** | **1** | **2** | **3** | **4** | **5** | **6** | **7** | **8** | **9** | **10** | **Summary** |
| --- | --- | --- | --- | --- | --- | --- | --- | --- | --- | --- | --- | --- | --- |
| Backett-Milburn  2003 [1] | Interview | 15 | Y | Y | Y | Y | Y | N | U | U | Y | Little data relating to HI but some evidence of resilience | Appropriate method but no reflexivity and unclear analysis. |
| Bowyer  2015 [2] | Interview | 5 | Y | Y | Y | Y | Y | N | Y | Y | Y | Lots of relevant data, but on HI in the context of domestic violence and relevant trauma | Appropriate method and analysis, good consideration of ethics, no reflexivity. |
| Bradley  2020 [3] | Interview (mixed methods evaluation of intervention) | 13 | Y | Y | Y | Y | Y | N | U | Y | Y | Some evidence relating to wellbeing, but mostly through child behaviour | Appropriate method but no reflexivity and unclear reporting of ethical issues (ethics approval granted but issues not discussed). |
| Jolly  2018 [4] | Interview | 15 | Y | Y | Y | Y | Y | N | Y | U | Y | Useful to have representation from this participant group. | Clear aims and methods, no reflexivity, extrapolation from data not completely clear, few quotations. |
| Karim  2006 [5] | Longitudinal, interview (mixed methods) | 35 | Y | Y | Y | Y | Y | N | Y | U | Y | Longitudinal aspect useful, however data reported is not very in-depth. | Clear aims and methods, no reflexivity. Analysis by questions. |
| Lawson  2015 [6] | Longitudinal, interview | 14 | Y | Y | Y | Y | Y | N | Y | Y | Y | A useful example of research relating to relocation, longitudinal nature also valuable | Clear aims and methods, no reflexivity |
| Lawson  2016 [7] | Longitudinal, interview | 10 (families) | Y | Y | Y | Y | U | N | Y | U | Y | A useful example of research relating to relocation, longitudinal nature also valuable | Data collection not optimal for research issue as was a post-hoc analysis of existing data. No reflexivity. Little detail on analysis. Ethics outlined but Lawson (2015) (same study) states no ethical approval to interview CYP. |
| Moffatt  2016 [8] | Interviews & focus groups | 38 + 12 | Y | Y | Y | Y | Y | N | Y | Y | Y | Not much on HI in families, but bedroom tax aspect is useful | Clear aims and methods, no reflexivity |
| Nettleton 2000 [9] | Interview | 44+  17 | Y | Y | Y | U | Y | N | U | Y | Y | HI but related to mortgage repossession. So a subgroup. | Very little data on methods or analysis. No reflexivity. |
| Oldman 2000 [10] | Interviews (in depth) | 40 | N | Y | Y | Y | Y | N | N | Y | Y | Limited data on housing instability. | Sample size unclear. No reflexivity. |
| Rowley 2020 [11] | Interviews | 9 | Y | Y | Y | Y | Y | Y | U | Y | Y | No details on children. | Limited relevant data. Well written |
| Thompson  2017 [12] | Narrative family interviews & ‘go-along’ interviews | 40 | Y | Y | Y | Y | Y | N | Y | Y | Y |  | Appropriate method but no reflexivity |
| Tischler 2007 [13] | Semi-structured interviews | 28 | Y | Y | Y | Y | Y | Y | Y | Y | Y | Lots regarding impact on mothers’ mental health – not explicitly related to impact on children | Ethical approval but v. little detail re. ethical issues |
| Tischler 2004 [14] | Qual element = semi-structured interviews | 49 | Y | Y | Y | Y | Y | N | Y | Y | Y | Relevant insights regarding children’s mental health and related needs | Appropriate method but no reflexivity |
| Tod 2015 [15] | Semi-structured interviews | 50 | Y | Y | Y | Y | Y | N | Y | Y | Y | Focus on fuel poverty but useful in highlighting interaction between fuel poverty and housing insecurity. |  |
| Warfa 2006 [16] | Group discussions | 34 | Y | Y | Y | Y | Y | N | Y | Y | Y | useful insights regarding interrelationship between residential instability, past traumatic experiences, homelessness BUT hard to discern impact on individuals / families / children | Appropriate method but no reflexivity |
| Watt  2018 [17] | Interview and observation | 17 | N | Y | Y | Y | Y | N | N | N | N | Useful in highlighting challenges of housing insecurity from mothers’ perspective |  |
| Wilcox 2000 [18] | In-depth interviews and participant observation | 20 | Y | Y | Y | Y | Y | N | N | N | Y | Some insights into impact on children of financial hardships experienced by mothers in the study. | Appropriate method but no reflexivity and no clear analysis of ethical issues. |

*Abbreviations: N=, Sample size; N, no; U, unclear; Y, yes.*

Checklist questions:

1. Was there a clear statement of the aims of the research?

2. Is a qualitative methodology appropriate?

3. Was the research design appropriate to address the aims of the research?

4. Was the recruitment strategy appropriate to the aims of the research?

5. Was the data collected in a way that addressed the research issue?

6. Has the relationship between researcher and participants been adequately considered?

7. Have ethical issues been taken into consideration?

8. Was the data analysis sufficiently rigorous?

9. Is there a clear statement of findings?

10. How valuable is the research?

**Supplementary Table 4: Quality appraisal of grey literature using the Authority, Accuracy, Coverage, Objectivity, Date, Significance (AACODS) checklist**

| **First author / year** | **Design** | **N=** | **1** | **2** | **3** | **4** | **5** | **6** | **Summary** |
| --- | --- | --- | --- | --- | --- | --- | --- | --- | --- |
| Minton  2005 [19] | Meetings and conversations | Nearly 50 | Y | U | Y | U | Y | Y | Clear aim but some methods unclear, including recruitment, data collection and analysis. ‘Objectivity’ is unclear although some degree of subjectivity should be expected in qualitative research? Has significance. |
| The Children’s Commissioner  2021 [20] | Interviews and focus groups | NR | Y | N | U | U | Y | U | Little data from a broad report, data collection and analysis methods not reported, ‘objectivity’ is unclear although looks like the views of the children are the focus, unclear significance as the quotation is only short. |
| Children’s Commissioner  2020 [21] | Unclear. Consultations? | NR | Y | N | N | U | Y | N | Little data from a broad report, data collection and analysis methods not reported, ‘objectivity’ is unclear although looks like the views of the children are the focus, unclear significance as the quotations are sparse and short. |
| Children’s Commissioner  2019 [22] | Unclear – conversations? | NR | Y | N | Y | U | Y | Y | Clear aim but data collection and analysis methods not reported, ‘objectivity’ is unclear although looks like the views of the children are the focus, seems significant and highlights a range of health and wellbeing issues. |
| Children’s Commissioner  2017 [23] | Mosaic approach | 40 | Y | Y | Y | U | Y | Y | Methods reported, organisation is reputable, coverage clear, ‘objectivity’ is unclear although looks like the views of the children are the focus, date clear, significant as reports on the link between suitability of housing and HI. |
| Joshi  2015 [24] | Mosaic approach | 40 | Y | Y | Y | Y | Y | Y | Methods reported, organisation is reputable, coverage clear, ‘objectivity’ is unclear although looks like the views of the children are the focus, date clear, reports on same data as Children’s Commissioner 2017 but with greater detail and nuance. |
| Shelter 2014 [25] | Qualitative interviews | 171 | Y | Y | Y | Y | Y | Y | Reputable organisation with named authors. Reflexivity not overtly stated. Analysis clearly presented and detailed quotes. No reflexivity. |
| Shelter 2018 [26] | Mixed methods | NR | Y | Y | Y | Y | N | Y | No methodology. Date unclear. No detail on study participants. No reflexivity. |
| Shelter 2021 [27] | Press release | NR | Y | N | Y | N | Y | N | Press release. Limited. Potential for bias. No reflexivity. |
| Shelter 2017a [28] | Qualitative interviews | 23:  11 kids | Y | Y | Y | Y | Y | Y | Well written qualitative report. No reflexivity. |
| Shelter 2012 [29] | Policy briefing | NR | Y | Y | Y | N | Y | Y | Evidence briefing. Risk of repeat data from other included reports. No reflexivity. |
| Shelter 2015 [30] | Qualitative interviews | 20 | Y | Y | Y | Y | Y | Y | Well written qualitative report. No reflexivity. |
| Shelter 2016b [31] | Qualitative interviews | 25 | Y | Y | Y | Y | Y | Y | Well written qualitative report. No reflexivity. |
| Shelter 2016c [32] | Qualitative interviews | 19 | Y | Y | Y | Y | Y | Y | Well written qualitative report. No reflexivity. |
| Shelter 2004b [33] | Qualitative interviews | 29 | Y | Y | Y | Y | Y | Y | Well written qualitative report. No reflexivity. |
| Shelter 2017b [34] | Qualitative interviews | 11 | Y | Y | Y | Y | Y | Y | Well written qualitative report. No reflexivity. |
| Shelter 2004c [35] | Qualitative interviews | Unclear | Y | Y | Y | Y | Y | Y | Size of interview sample unclear. No reflexivity. |
| Shelter 2004d [36] | Not stated (presume interviews for quote) | 1 | Y | N | Y | N | Y | Y | One relevant quote only, no methodology. No reflexivity. |
| Renter's Reform Coalition 2022 [37] | NR | NR | Y | N | Y | Y | Y | Y | No methodology, no description of who was interviewed. No reflexivity. |
| JRF 2018 [38] | Qualitative longitudinal | 72 | Y | Y | Y | Y | Y | Y | Complete methodology. Well written report. No reflexivity. |
| JRF 2021 [39] | Annual report | NR | Y | N | Y | Y | Y | N | Annual report. No methodology or defined sample. |
| JRF 2017 [40] | Qualitative interviews | 145 | Y | Y | Y | Y | Y | Y | Some information on methodology. Well written report. No reflexivity. |
| YWT 2020 [41] | Focus group | 4 | Y | Y | N | Y | Y | Y | Methodology in linked references. No detail on children. |
| White  2008 [42] | Case study plus interviews | 12 (+44) | Y | Y | Y | Y | Y | U | Not much relevant info to extract, and no relevant data, but useful to have some evidence relating to an (holistic) intervention. |
| Hardy and Gillespie 2016 [43] | Structured interviews | 32 | Y | N | Y | N | Y | Y | Quoatations not linked to narrative. No reflexivity. Aim not clearly stated. |
| Dexter 2016  (The Children’s Society) [44] | Interviews, case studies | 8 | Y | Y | Y | Y | N | Y | Clear aim, methods of analysis unclear but other methods clear, has significance in terms of population. |
| Price  2015 [45] | Interviews | 91 | Y | Y | Y | Y | Y | Y | Clear aim, methods of analysis unclear but other methods clear, has significance in terms of population. |
| Coram Children’s Legal Centre  2013 [46] | Case studies | NR | Y | N | Y | Y | Y | Y | Methods lacking in detail, no detail on sample, or on how case studies were selected. Population significant. |
| Children’s Society  2020 [47] | Interviews, case studies | 24 | Y | Y | Y | Y | Y | Y | High quality, in-depth research relating to HI across the country. |
| Children’s Society  2020  (Pinter 2020) [48] | Interviews | 11 | Y | U | Y | Y | Y | Y | Clear aim & data collection, analysis not clear. Has significance. |
| Children’s Society  2017 [49] | Interviews, focus groups | 60 | Y | Y | Y | Y | Y | Y | Same sample/data collection as Children’s Society 2020 (Moving, Always Moving). Good quality, academic, adds primary data on related themes. A lot of detail on HI. |
| CPAG & CoE (2020) [50] | Interviews | 21 | Y | Y | Y | Y | Y | Y | Not much re housing |
| CPAG (2020) [51] | Narrative element of survey | 124 | Y | Y | Y | Y | Y | Y | Well written report. Helpful perspective of social workers re. homelessness / housing insecurity. |
| RCPCH (2017) Poverty & Us [52] | workshop | number unclear | Y | N | N | N | Y | N | V. little detail / context to quotes. |
| RCPCH (2017) Poverty and child health: views from the frontline [53] | survey open text responses | 266 | Y | Y | Y | Y | Y | Y | v little detail re, methodology |
| Project 17  2019 [54] | Interviews, workshop, open-ended survey | 17 (int) / 14 (wshp) | Y | U | Y | Y | N | Y | Clear aim & data collection, analysis not clear, no date given. Has significance. |
| Project 17  2018 [55] | Case studies, open-ended survey | Unclear (2 families) | Y | U | Y | Y | Y | N | Recruitment, data collection and analysis not clear – seems more informal and illustrative. Data includes nothing new and not much on the impact on the children. |
| Office of the Deputy Prime Minister  2005 [56] | Interviews | 82 house-holds | Y | Y | Y | Y | Y | Y | Important info on the link between requesting repairs (for poor quality accommodation that was impacting children’s health) and eviction. |
| Scottish Women’s Aid  2015 [57] | Interviews, open-ended survey responses | 4 (int),  45 (survey) | Y | U | Y | Y | Y | Y | Recruitment to interviews, data collection and analysis not clear. Participatory approach useful. |
| Jones  2010 [58] | Interview | 114 | Y | Y | Y | Y | Y | Y | Clear aim and methods. Useful as examines keeping families in the same home following DV. |
| Maternity Action  2022 [59] | Online group discussion | 10 | Y | U | Y | Y | N | Y | Clear aims. No details on analysis. No dates. Some useful data. |

*Abbreviations: CPAG, Child Poverty Action Group; CoE, Church of England; JRF, Joseph Rowntree Foundation; N=, Sample size; N, no; RCPCH, Royal College of Paediatrics and Child Health; U, unclear; Y, yes; YWT, Young Women’s Trust.*

Checklist questions:

1. Authority (Y, N, U) – consider:

Identifying who is responsible for the intellectual content.

- Individual author:
  - Associated with a reputable organisation?
  - Professional qualifications or considerable experience?
  - Produced/published other work (grey/black) in the field?
  - Recognised expert, identified in other sources?
  - Cited by others? (use Google Scholar as a quick check)
  - Higher degree student under “expert” supervision?
- Organisation or group:
  - Is the organisation reputable? (e.g. W.H.O)
  - Is the organisation an authority in the field?
- In all cases:
  - Does the item have a detailed reference list or bibliography?

2. Accuracy (Y, N, U) – consider:

- Does the item have a clearly stated aim or brief?
- Is so, is this met?
- Does it have a stated methodology?
- If so, is it adhered to?
- Has it been peer-reviewed?
- Has it been edited by a reputable authority?
- Supported by authoritative, documented references or credible sources?
- Is it representative of work in the field?
- If No, is it a valid counterbalance?
- Is any data collection explicit and appropriate for the research?
- if item is secondary material (e.g. a policy brief of a technical report) refer to the original. Is it an accurate, unbiased interpretation or analysis?

3. Coverage (Y, N, U) – consider:

All items have parameters which define their content coverage. These limits might mean that a work refers to a particular population group, or that it excluded certain types of publication. A report could be designed to answer a particular question, or be based on statistics from a particular survey.

- Are any limits clearly stated?

4. Objectivity (Y, N, U) – consider:

It is important to identify bias, particularly if it is unstated or unacknowledged.

- Opinion, expert or otherwise, is still opinion: is the author’s standpoint clear?
- Does the work seem to be balanced in presentation?

5. Date (Y, N, U) – consider:

For the item to inform your research, it needs to have a date that confirms relevance

- Does the item have a clearly stated date related to content? No easily discernible date is a strong concern.
- If no date is given, but can be closely ascertained, is there a valid reason for its absence?
- Check the bibliography: have key contemporary material been included?

6. Significance (Y, N, U) – consider:

This is a value judgment of the item, in the context of the relevant research area

- Is the item meaningful? (this incorporates feasibility, utility and relevance)
- Does it add context?
- Does it enrich or add something unique to the research?
- Does it strengthen or refute a current position?
- Would the research area be lesser without it?
- Is it integral, representative, typical?

Does it have impact? (in the sense of influencing the work or behaviour of others)

**References**

1. Backett-Milburn K, Cunningham-Burley S, Davis J: **Contrasting lives, contrasting views? understandings of health inequalities from children in differing social circumstances.** *Social Science & Medicine* 2003, **57:**613-623.

2. Bowyer L, Swanston J, Vetere A: **'Eventually you just get used to it': An interpretative phenomenological analysis of 10-16 year-old girls' experiences of the transition into temporary accommodation after exposure to domestic violence perpetrated by men against their mothers.** *Clinical Child Psychology and Psychiatry* 2015, **20:**304-323.

3. Bradley C, Day C, Penney C, Michelson D: **'Every day is hard, being outside, but you have to do it for your child': Mixed-methods formative evaluation of a peer-led parenting intervention for homeless families.** *Clinical Child Psychology and Psychiatry* 2020, **25:**860-876.

4. Jolly A: **No Recourse to Social Work? Statutory Neglect, Social Exclusion and Undocumented Migrant Families in the UK.** *Social Inclusion* 2018, **6:**190-200.

5. Karim K, Tischler V, Gregory P, Vostanis P: **Homeless children and parents: Short-term mental health outcome.** *International Journal of Social Psychiatry* 2006, **52:**447-458.

6. Lawson L, Kearns A, Egan M, Conway E: **"You Can't Always Get What You Want... "? Prior-Attitudes and Post-Experiences of Relocation from Restructured Neighbourhoods.** *Housing Studies* 2015, **30:**942-966.

7. Lawson L, Kearns A: **'Power to the (young) people'? Children and young people's empowerment in the relocation process associated with urban re-structuring.** *International Journal of Housing Policy* 2016, **16:**376-403.

8. Moffatt S, Lawson S, Patterson R, Holding E, Dennison A, Sowden S, Brown J: **A qualitative study of the impact of the UK 'bedroom tax'.** *Journal of Public Health* 2016, **38:**197-205.

9. Nettleton S, Burrows R: **When a capital investment becomes an emotional loss: The health consequences of the experience of mortgage possession in England.** *Housing Studies* 2000, **15:**463-479.

10. Oldman C, Beresford B: **Home, sick home: Using the housing experiences of disabled children to suggest a new theoretical framework.** *Housing Studies* 2000, **15:**429-442.

11. Rowley L, Morant N, Katona C: **Refugees Who Have Experienced Extreme Cruelty: A Qualitative Study of Mental Health and Wellbeing after Being Granted Leave to Remain in the UK.** *Journal of Immigrant & Refugee Studies* 2020, **18:**357-374.

12. Thompson C, Lewis DJ, Greenhalgh T, Smith NR, Fahy AE, Cummins S: **"I don't know how I'm still standing" a Bakhtinian analysis of social housing and health narratives in East London.** *Social Science and Medicine* 2017, **177:**27-34.

13. Tischler V, Rademeyer A, Vostanis P: **Mothers experiencing homelessness: mental health, support and social care needs.** *Health & Social Care in the Community* 2007, **15:**246-253.

14. Tischler V, Karim K, Rustall S, Gregory P, Vostanis P: **A family support service for homeless children and parents: users' perspectives and characteristics.** *Health & Social Care in the Community* 2004, **12:**327-335.

15. Tod AM, Nelson P, De Chavez AC, Homer C, Powell-Hoyland V, Stocks A: **Understanding influences and decisions of households with children with asthma regarding temperature and humidity in the home in winter: A qualitative study.** *BMJ Open* 2016, **6:**e009636.

16. Warfa N, Bhui K, Craig T, Curtis S, Mohamud S, Stansfeld S, McCrone P, Thornicroft G: **Post-migration geographical mobility, mental health and health service utilisation among Somali refugees in the UK: A qualitative study.** *Health & Place* 2006, **12:**503-515.

17. Watt P: **Gendering the right to housing in the city: Homeless female lone parents in post-Olympics, austerity East London.** *Cities* 2018, **76:**43-51.

18. Wilcox P: **Lone motherhood: the impact on living standards of leaving a violent relationship.** *Social Policy & Administration* 2000, **34:**176-190.

19. Minton A, Jones S: **Generation squalor: Shelter’s national investigation into the housing crisis.** London: Shelter; 2005.

20. Children's Commissioner for England: **The big ask, the big answer.** London: Children's Commissioner for England; 2021.

21. Children's Commissioner: **"Are we there yet?" Our rights, our say: A report for the UN Committee on the Rights of the Child.** London: Children and Young People’s Commissioner Scotland, Children’s Commissioner for Wales, Northern Ireland Commissioner for Children and Young People, and Children’s Commissioner for England.; 2020.

22. Children’s Commissioner for England: **Bleak houses: Tackling the crisis of family homelessness in England.** London: Children’s Commissioner for England; 2019.

23. Children’s Commissioner for England: **Changing the odds in the early years.** London: Children’s Commissioner for England; 2017.

24. Joshi P, Wallace E, Williams L: **Young children’s and families’ experiences of services aimed at reducing the impact of low-income: Participation work with children and families.** London: Office of the Children’s Commissioner; 2015.

25. Shelter: **A Roof Over My Head: The final report of the Sustain project.** London: Shelter; 2014.

26. Shelter: **Building our future: A vision for social housing.** London: Shelter; 2018.

27. **Health of one in five renters harmed by their home.** [<https://england.shelter.org.uk/media/press_release/health_of_one_in_five_renters_harmed_by_their_home>]

28. Shelter: **‘We’ve got no home’: The experiences of homeless children in emergency accommodation.** London: Shelter; 2017.

29. Shelter: **Policy: Briefing. Homes fit for families? The case for stable private renting.** London: Shelter; 2012.

30. Shelter: **‘This is no place for a child’: the experiences of homeless families in emergency accommodation.** London: Shelter; 2015.

31. Shelter: **Desperate to escape: the experiences of homeless families in emergency accommodation.** London: Shelter; 2016.

32. Shelter: **The experiences of people in housing debt.** London: Shelter; 2016.

33. Shelter: **Listen up: The voices of homeless children.** London: Shelter; 2004.

34. Shelter: **Impacts of homelessness on children – research with teachers.** London: Shelter; 2017.

35. Shelter: **Sick and tired: The impact of temporary accommodation on the health of homeless families.** London: Shelter; 2004.

36. Shelter: **Toying with their future: The hidden cost of the housing crisis.** London: Shelter; 2004.

37. Renters' Reform Coalition: **Safe, secure and affordable homes for all: A renters’ blueprint for reform.**: Renters' Reform Coalition; 2022.

38. Croucher K, Quilgars D, Dyke A: **Housing and life experiences: making a home on a low income.** York: Joseph Rowntree Foundation; 2018.

39. Joseph Rowntree Foundation: **UK Poverty 2020/21: The leading independent report.** York: Joseph Rowntree Foundation; 2021.

40. Clarke A, Hamilton C, Jones M, Muir K: **Poverty, evictions and forced moves.** York: Joseph Rowntree Foundation; 2017.

41. Young Women's Trust: **On the edge: Life for young women on low incomes in London.** London: Young Women's Trust; 2020.

42. White C, Warrener M, Reeves A, La Valle I: **Family Intervention Projects: An Evaluation of their Design, Set-up and Early Outcomes.** London: Department for Children, Schools and Families; 2008.

43. Hardy K, Gillespie T: **Homelessness, health and housing: Participatory action research in East London.** Lancaster: The Sociological Review Foundation; 2016.

44. Dexter Z, Capron L, Gregg L: **Making Life Impossible: How the needs of destitute migrant children are going unmet.** London: The Children's Society; 2016.

45. Price J, Spencer S: **Safeguarding children from destitution: Local authority responses to families with 'no recourse to public funds'.** Oxford: Centre on Migration, Policy and Society, University of Oxford; 2015.

46. Coram Children's Legal Centre: **Growing Up In A Hostile Environment: The rights of undocumented migrant children in the UK.** London: Coram Children’s Legal Centre; 2013.

47. The Children's Society: **Moving, Always Moving: The normalisation of housing insecurity among children in low income households in England.** London: The Children's Society; 2020.

48. The Children's Society: **A lifeline for all: Children and families with no recourse to public funds.** London: The Children's Society; 2020.

49. The Children's Society: **Understanding childhoods: Growing up in hard times.** London: The Children's Society; 2017.

50. Child Poverty Action Group and Church of England: **Poverty in the pandemic: the impact of coronavirus on lowincome families and children.** London: Child Poverty Action Group; 2020.

51. Child Poverty Action Group: **The safety net is gone: Understanding the impact of poverty on the lives of children and families in England: a survey of social workers.** London: Child Poverty Action Group; 2020.

52. Royal College of Paediatrics and Child Health: **Poverty &Us - parents, carers and young people tell us how poverty impacts them.** London: Royal College of Paediatrics and Child Health; 2017.

53. Royal College of Paediatrics and Child Health: **Poverty and child health: views from the frontline.** London: Royal College of Paediatrics and Child Health; 2017.

54. Project 17: **Not seen, not heard: Children's experiences of the hostile environment.** London: Project 17; 2019.

55. Project 17: **"In the night we didn't know where we were going": Project 17's Hotel Fund.** London: Project 17; 2018.

56. Office of the Deputy Prime Minister: **Causes of homelessness amongst ethnic minority populations.** London: Office of the Deputy Prime Minister; 2005.

57. Scottish Women's Aid: **Change, justice, fairness: "Why should we have to move everywhere and everything because of him?".** Edinburgh: Scottish Women's Aid; 2015.

58. Jones A, Bretherton J, Bowles R, Croucher K: **The effectiveness of schemes to enable households at risk of domestic violence to remain in their homes: Research report.** London: Department for Communities and Local Government; 2010.

59. Maternity Action: **Maternal health: Exploring the lived experiences of pregnant women seeking asylum.** London: Maternity Action; 2022.
